# Supplementary material for: Responding to Bias: Equipping Residents With Tools to Address Microaggressions
Source: MedEdPORTAL. 2024 Aug 6;20:11424. doi: 10.15766/mep_2374-8265.11424 (PMC11300577; doi:10.15766/mep_2374-8265.11424)
Supplement: Supplementary file 1 — Bias Response Toolkit.docxBias Response Workshop.pptxFacilitator Guide.docxPre- and Postworkshop Survey Questions.docx [file mep_2374-8265.11424-s001.zip › B. Bias Response Workshop.pptx]

## Slide 1
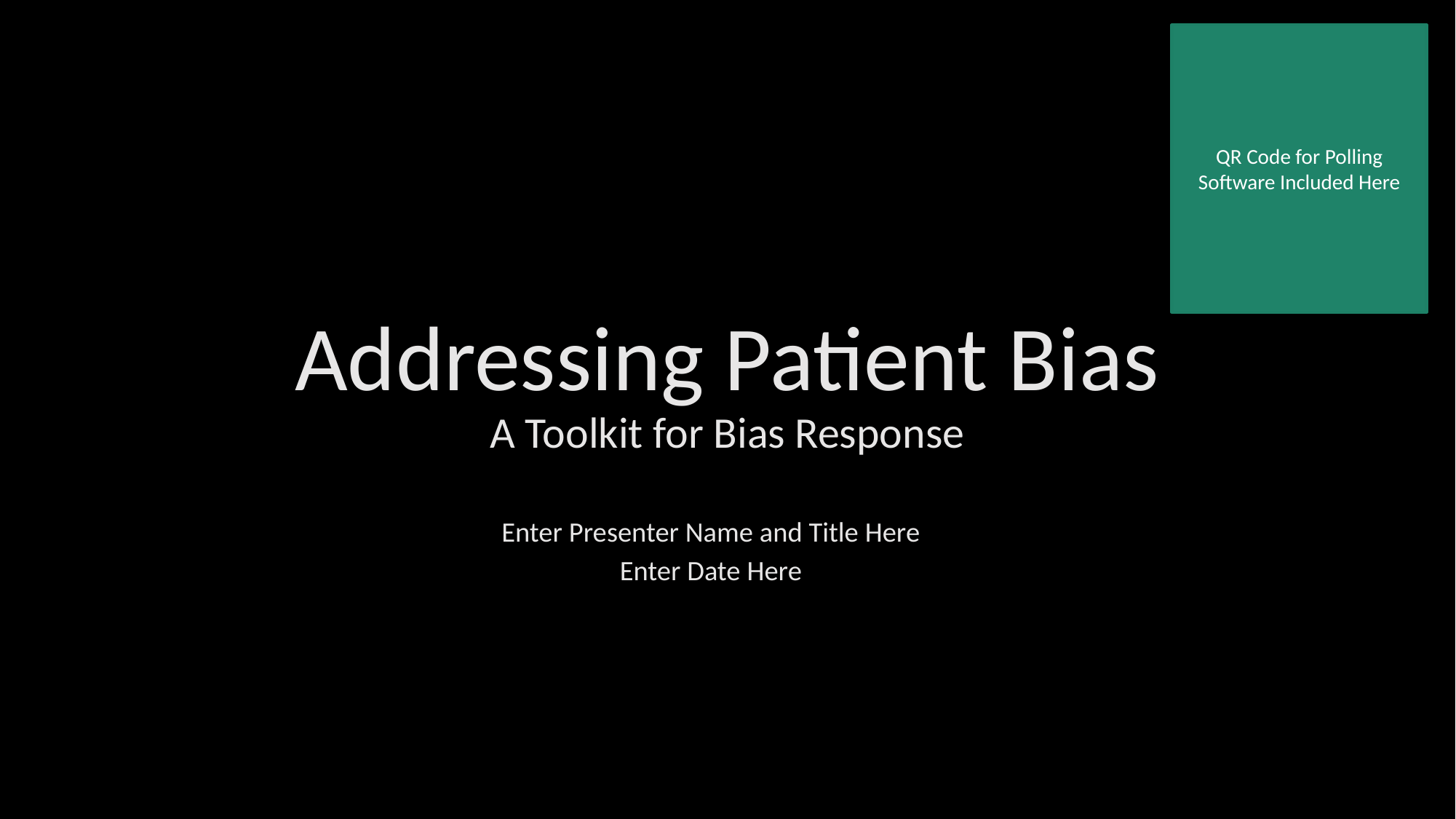

QR Code for Polling Software Included Here
Addressing Patient Bias
A Toolkit for Bias Response
Enter Presenter Name and Title Here
Enter Date Here

## Slide 2
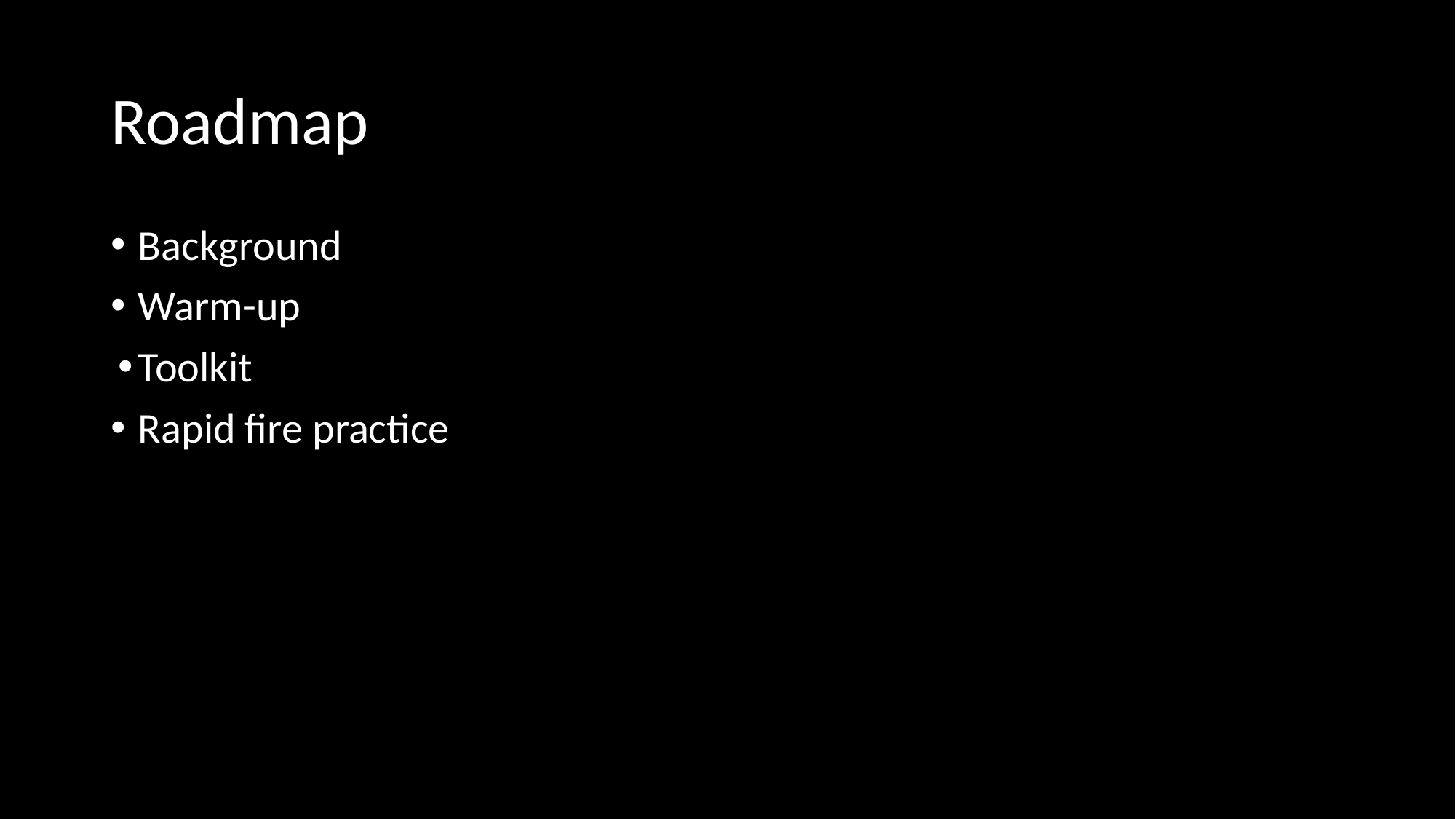

# Roadmap
Background
Warm-up
Toolkit
Rapid fire practice

## Slide 3
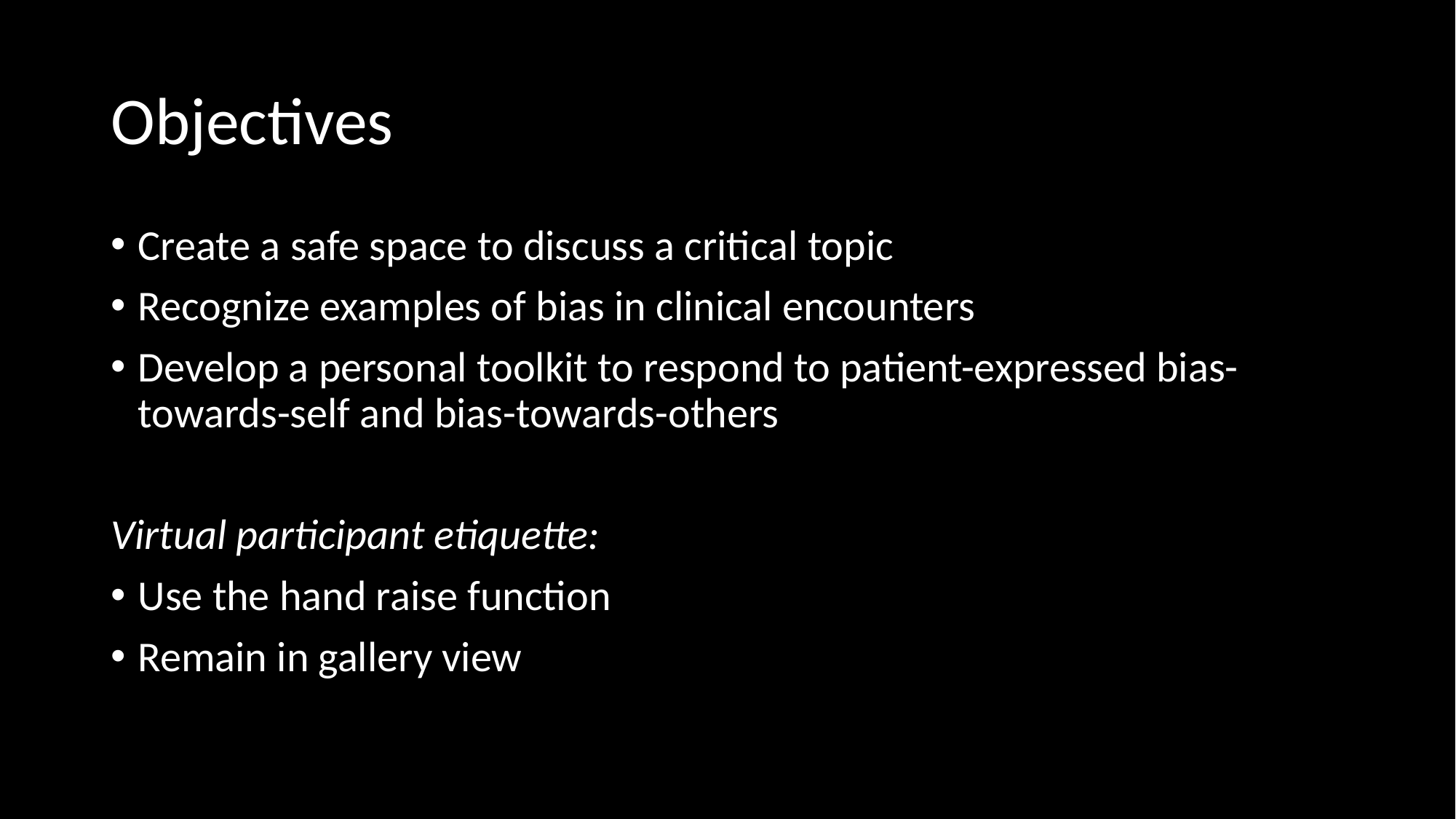

# Objectives
Create a safe space to discuss a critical topic
Recognize examples of bias in clinical encounters
Develop a personal toolkit to respond to patient-expressed bias-towards-self and bias-towards-others
Virtual participant etiquette:
Use the hand raise function
Remain in gallery view

## Slide 4
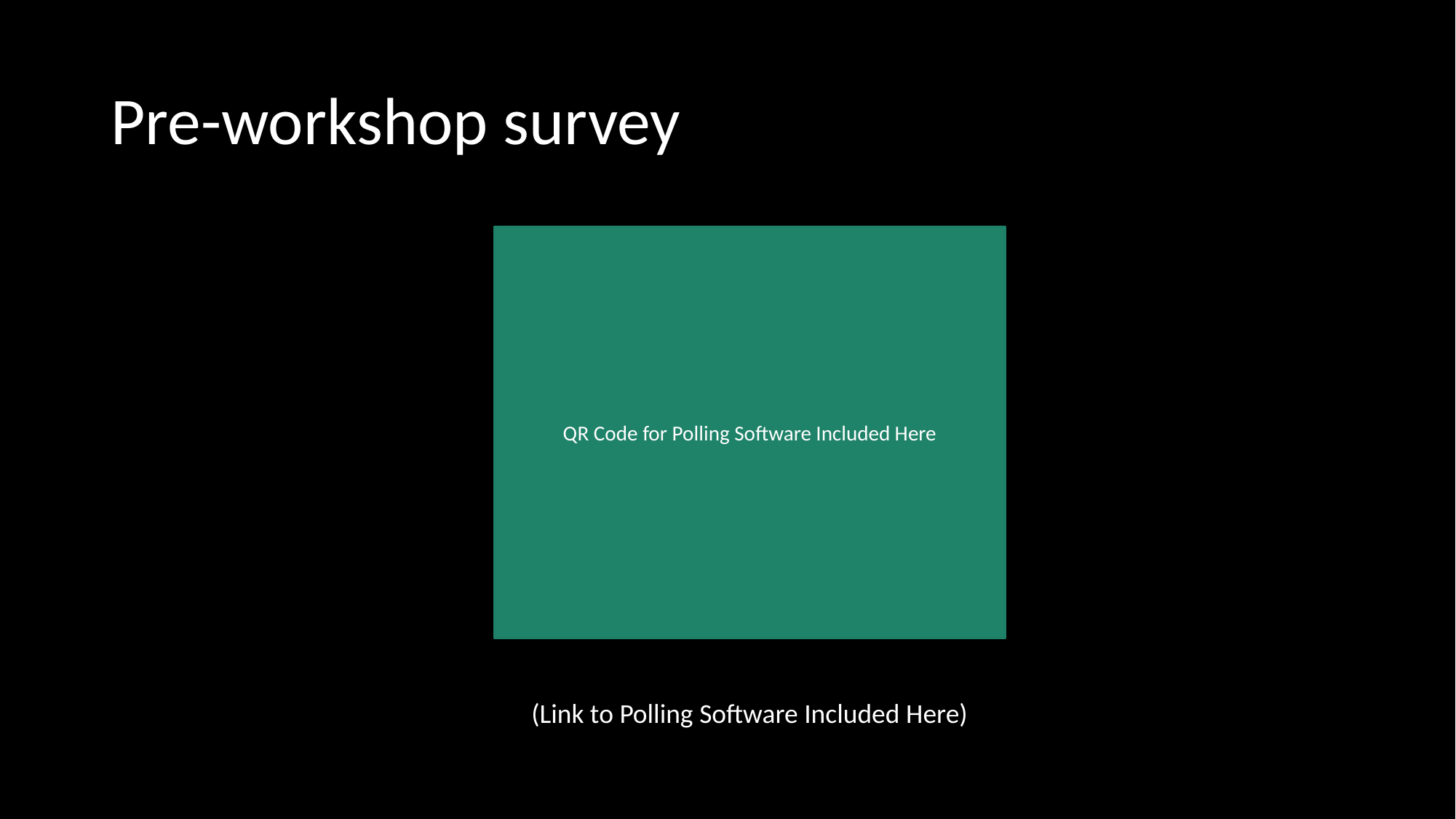

# Pre-workshop survey
QR Code for Polling Software Included Here
(Link to Polling Software Included Here)

## Slide 5
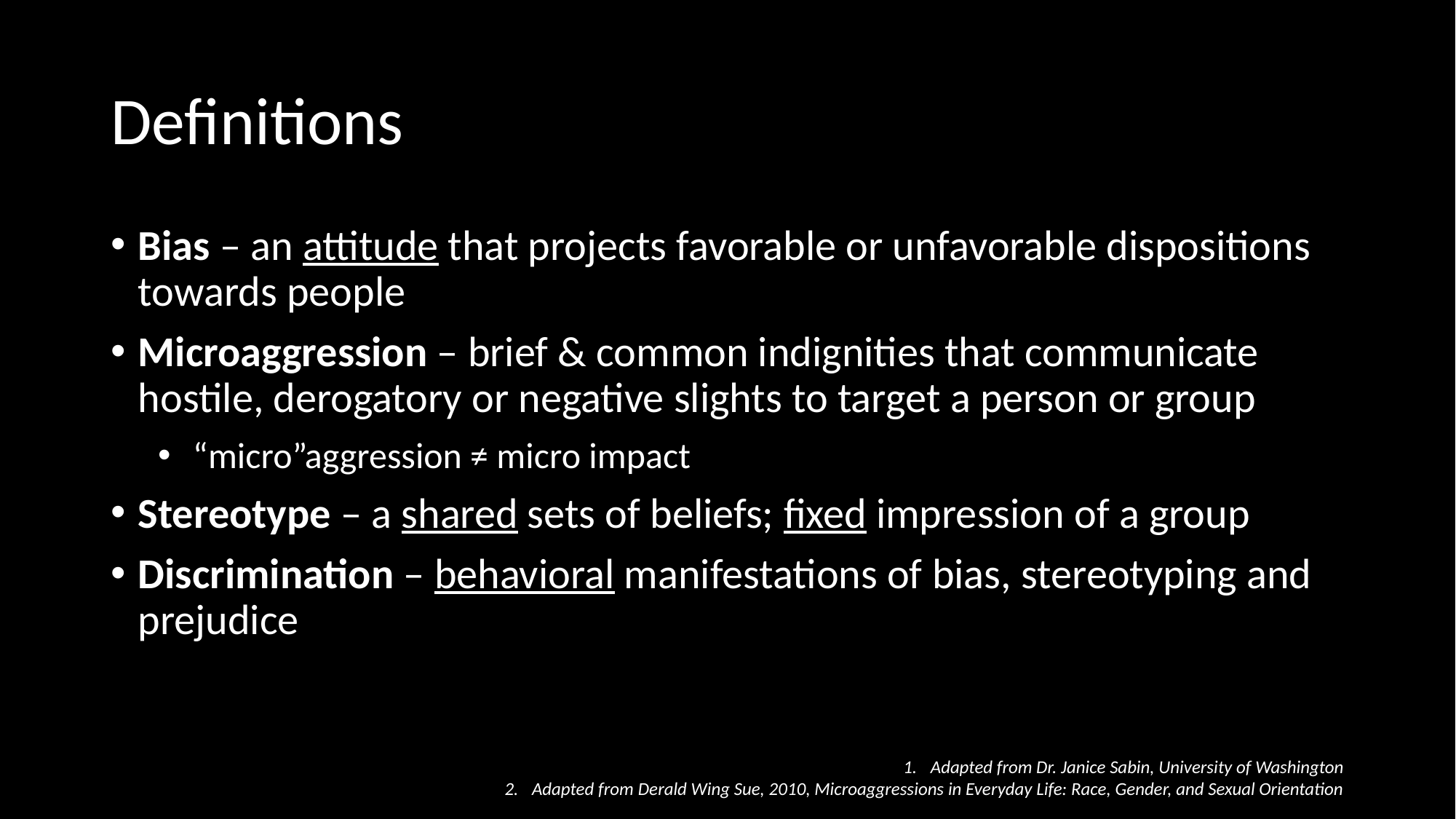

# Definitions
Bias – an attitude that projects favorable or unfavorable dispositions towards people
Microaggression – brief & common indignities that communicate hostile, derogatory or negative slights to target a person or group
“micro”aggression ≠ micro impact
Stereotype – a shared sets of beliefs; fixed impression of a group
Discrimination – behavioral manifestations of bias, stereotyping and prejudice
Adapted from Dr. Janice Sabin, University of Washington
Adapted from Derald Wing Sue, 2010, Microaggressions in Everyday Life: Race, Gender, and Sexual Orientation

## Slide 6
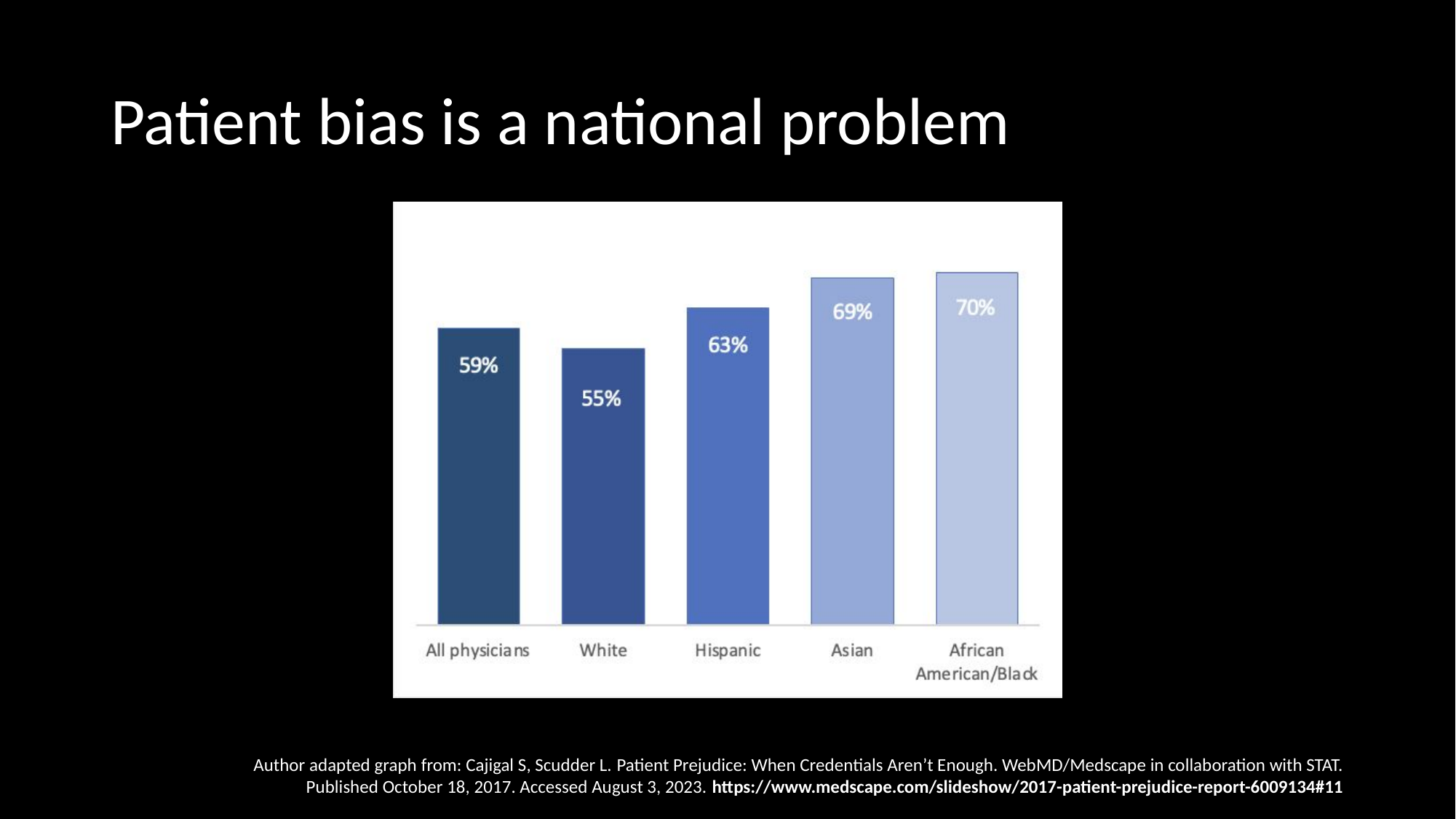

Patient bias is a national problem
Author adapted graph from: Cajigal S, Scudder L. Patient Prejudice: When Credentials Aren’t Enough. WebMD/Medscape in collaboration with STAT. Published October 18, 2017. Accessed August 3, 2023. https://www.medscape.com/slideshow/2017-patient-prejudice-report-6009134#11

## Slide 7
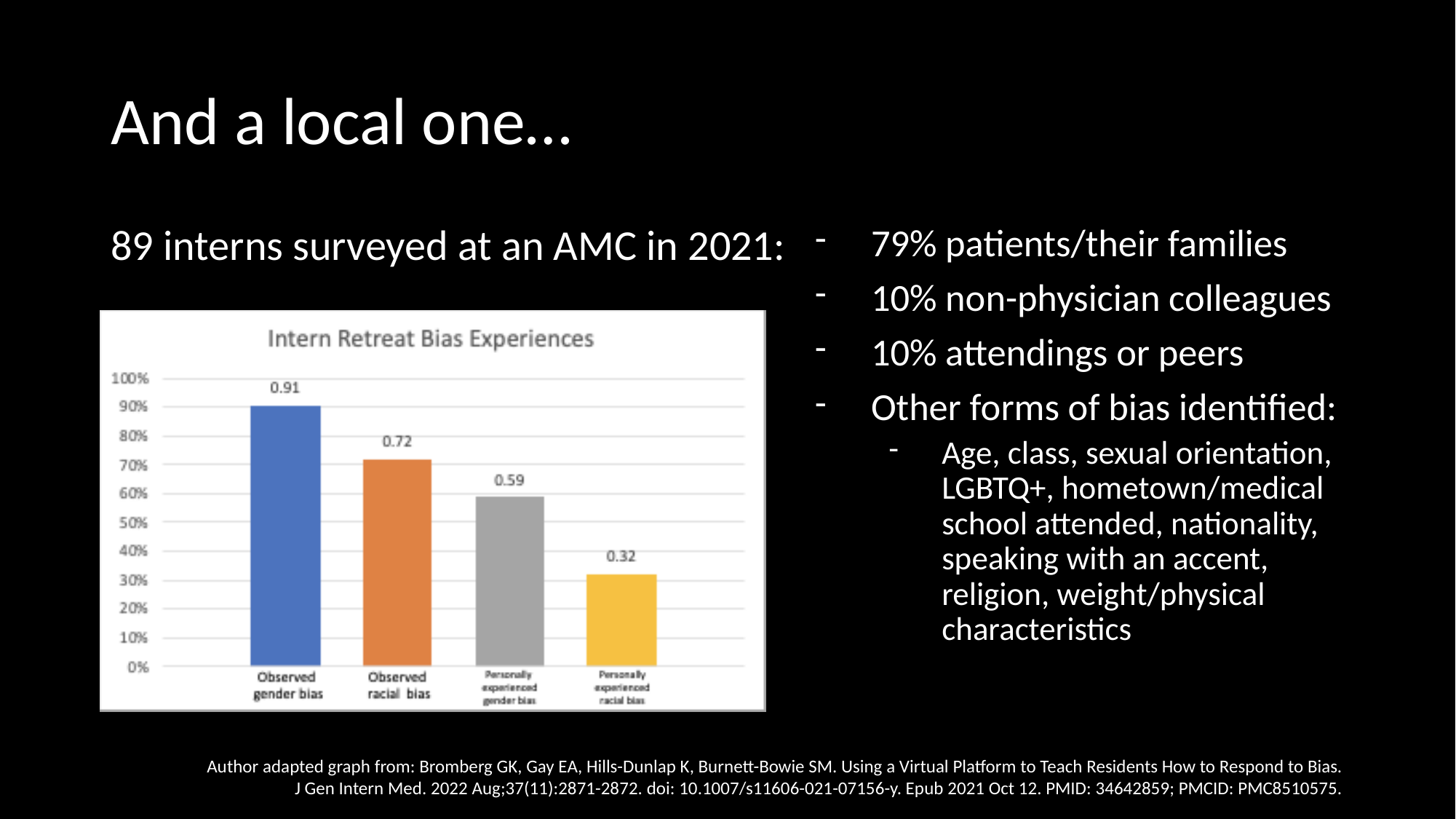

# And a local one…
89 interns surveyed at an AMC in 2021:
79% patients/their families
10% non-physician colleagues
10% attendings or peers
Other forms of bias identified:
Age, class, sexual orientation, LGBTQ+, hometown/medical school attended, nationality, speaking with an accent, religion, weight/physical characteristics
Author adapted graph from: Bromberg GK, Gay EA, Hills-Dunlap K, Burnett-Bowie SM. Using a Virtual Platform to Teach Residents How to Respond to Bias. J Gen Intern Med. 2022 Aug;37(11):2871-2872. doi: 10.1007/s11606-021-07156-y. Epub 2021 Oct 12. PMID: 34642859; PMCID: PMC8510575.

## Slide 8
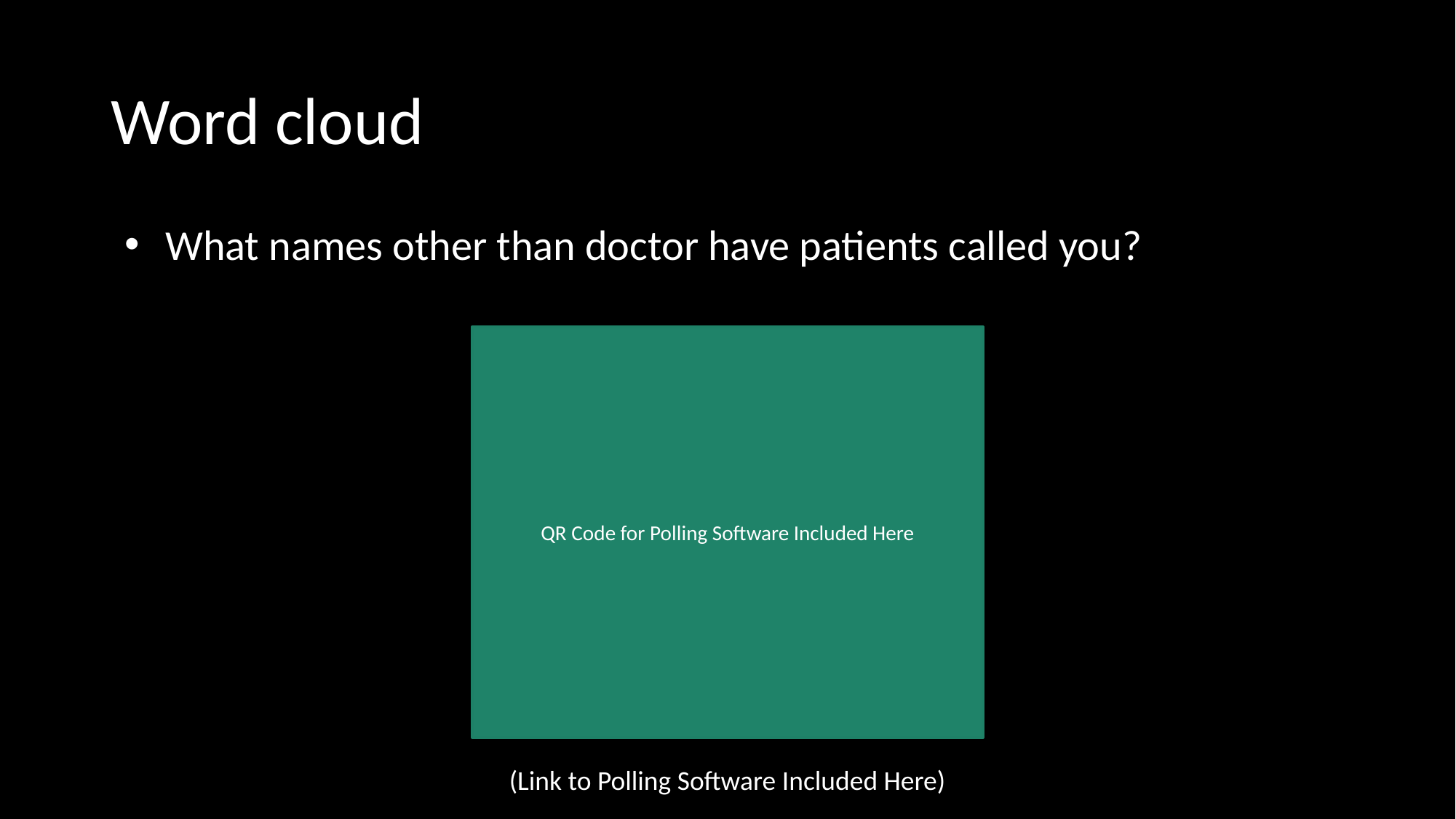

# Word cloud
What names other than doctor have patients called you?
QR Code for Polling Software Included Here
(Link to Polling Software Included Here)

## Slide 9
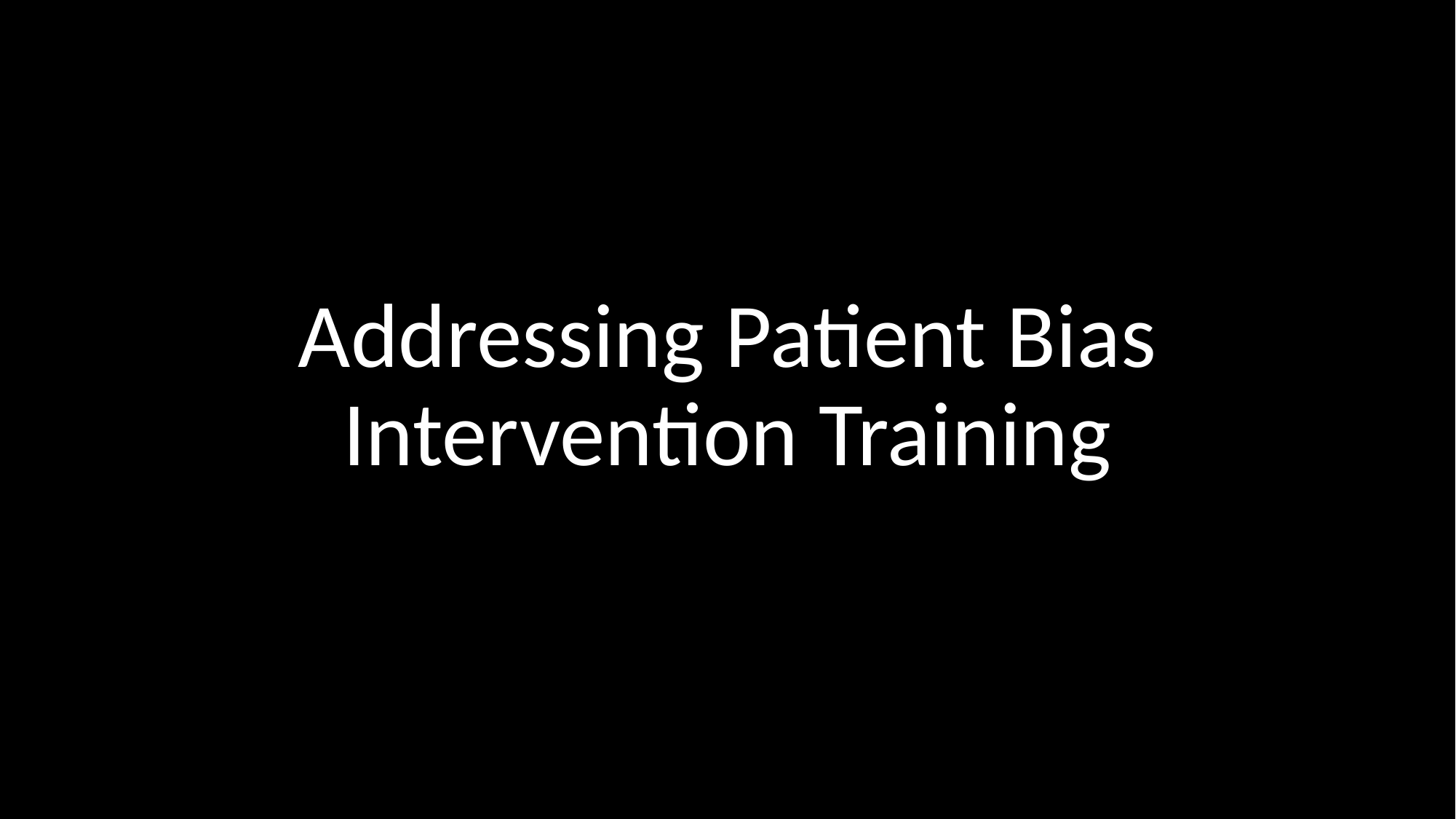

# Addressing Patient Bias Intervention Training

## Slide 10
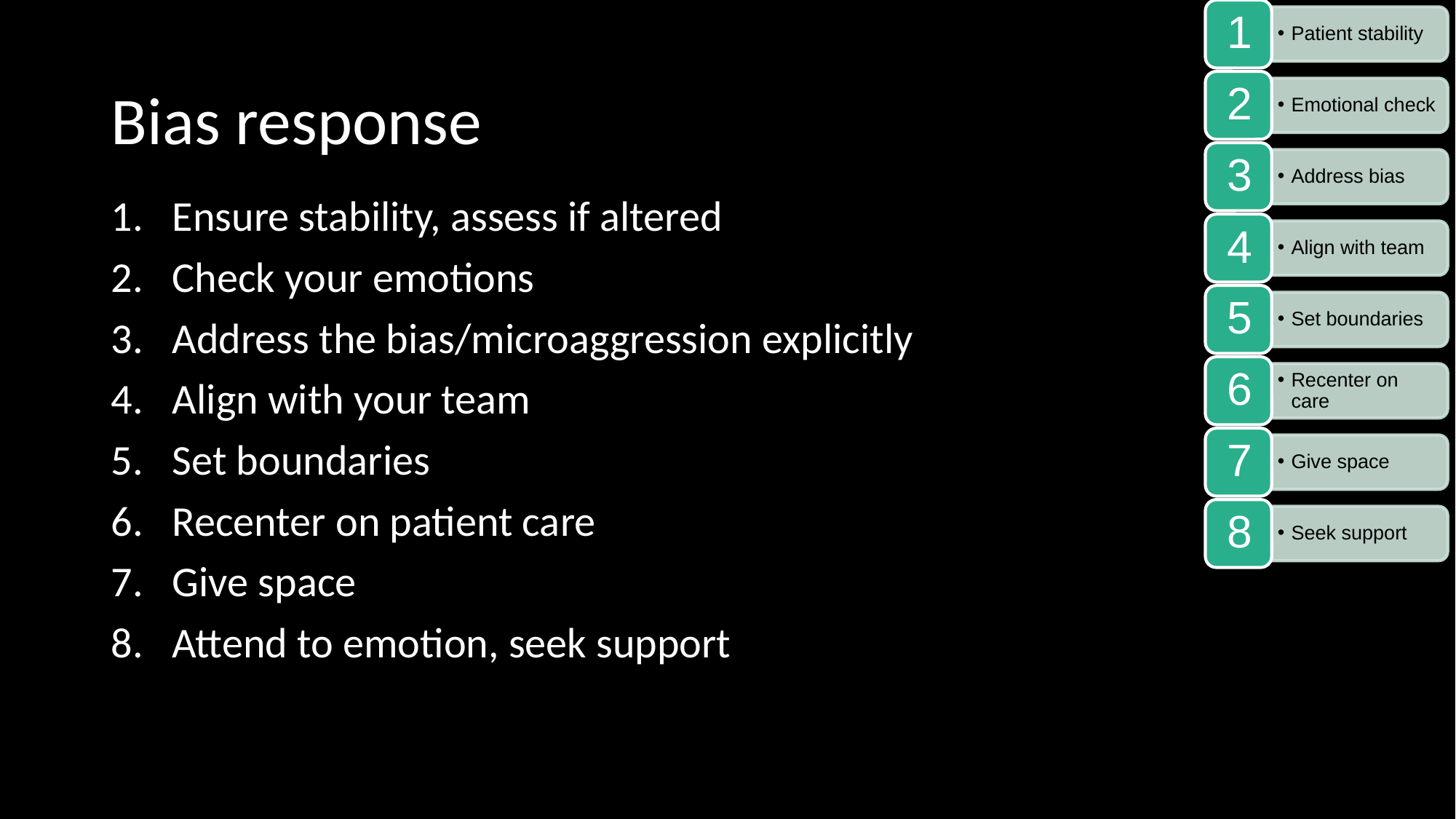

# Bias response
Ensure stability, assess if altered
Check your emotions
Address the bias/microaggression explicitly
Align with your team
Set boundaries
Recenter on patient care
Give space
Attend to emotion, seek support

## Slide 11
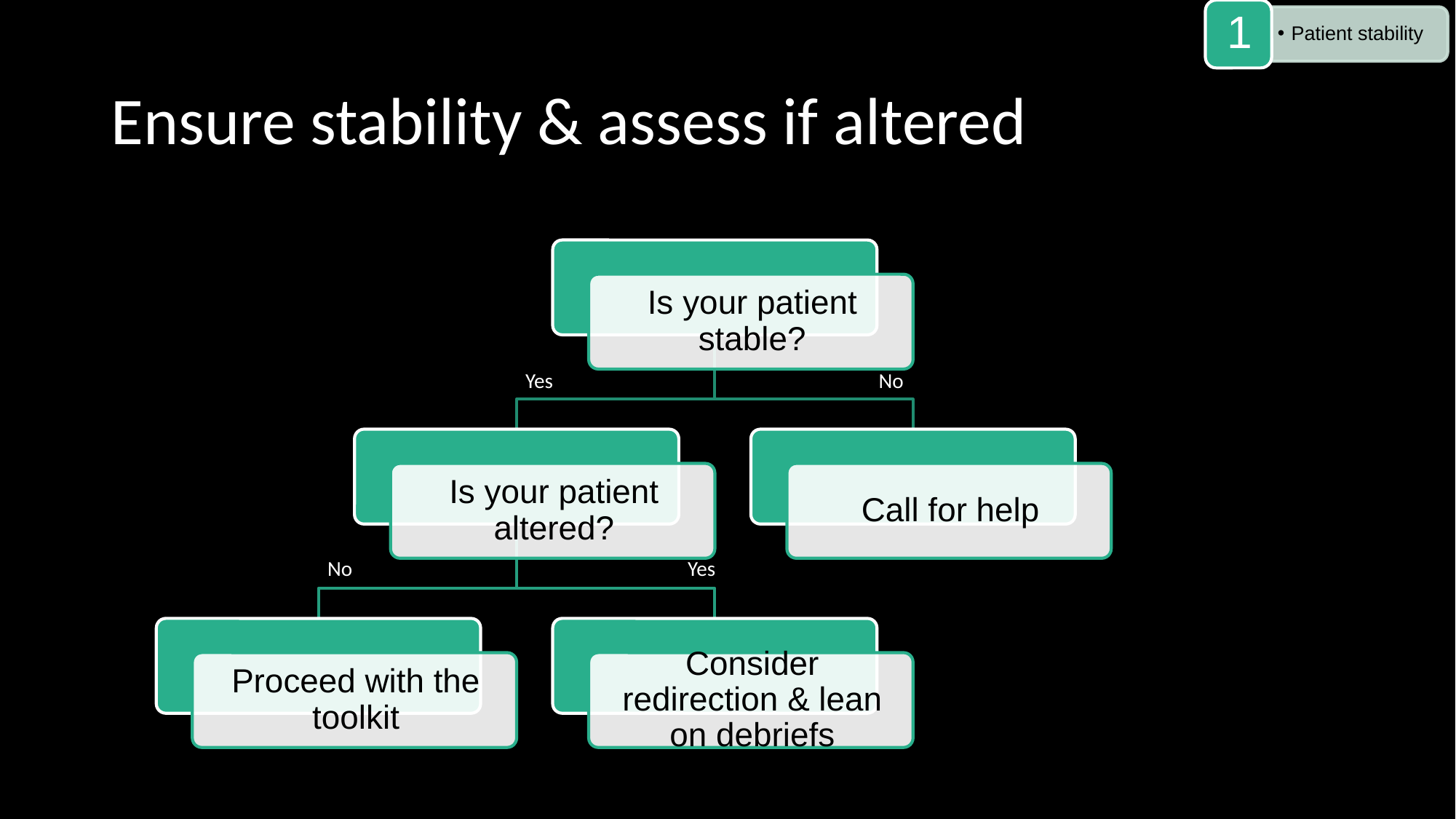

# Ensure stability & assess if altered
No
Yes
No
Yes

## Slide 12
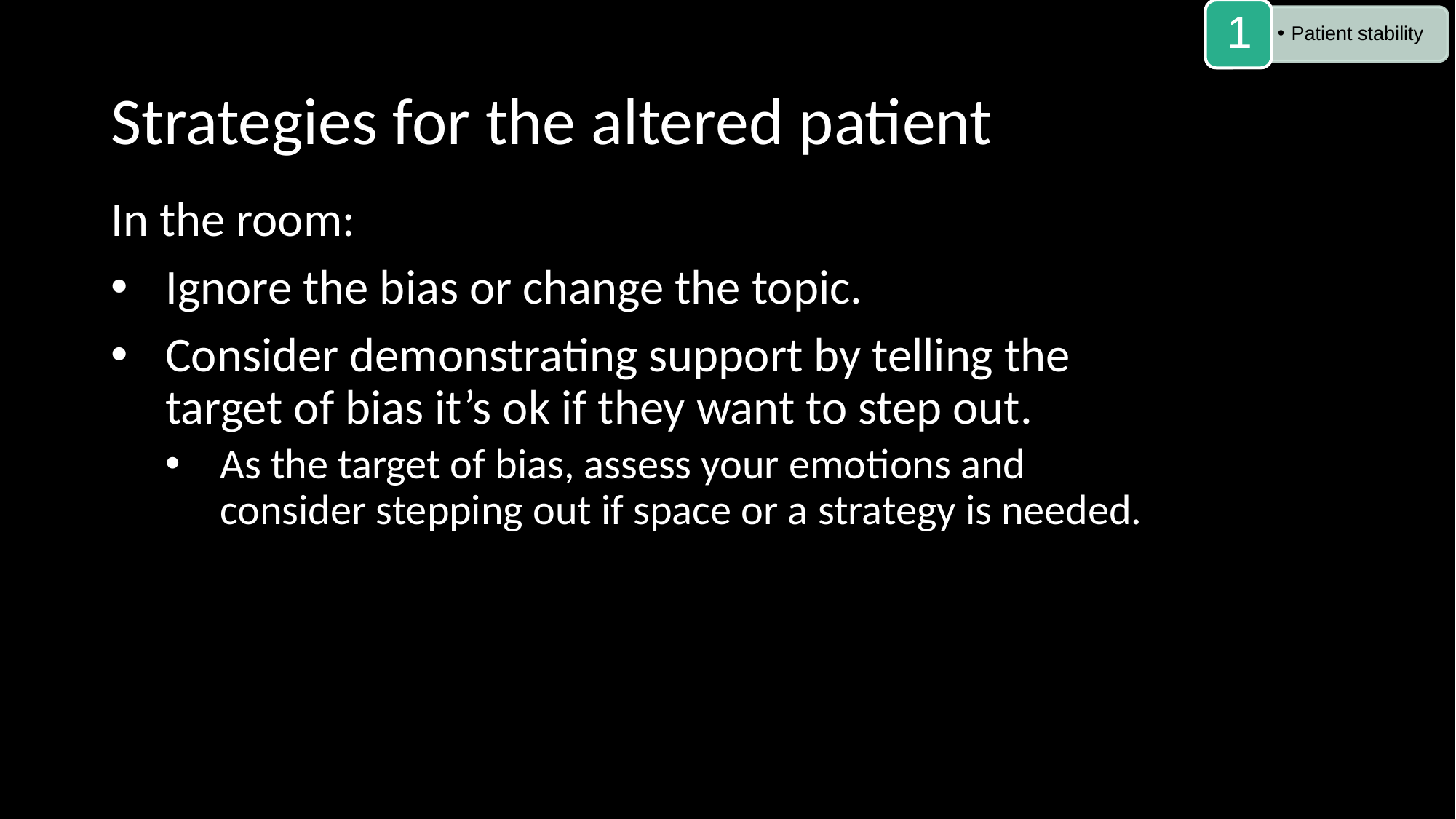

# Strategies for the altered patient
In the room:
Ignore the bias or change the topic.
Consider demonstrating support by telling the target of bias it’s ok if they want to step out.
As the target of bias, assess your emotions and consider stepping out if space or a strategy is needed.

## Slide 13
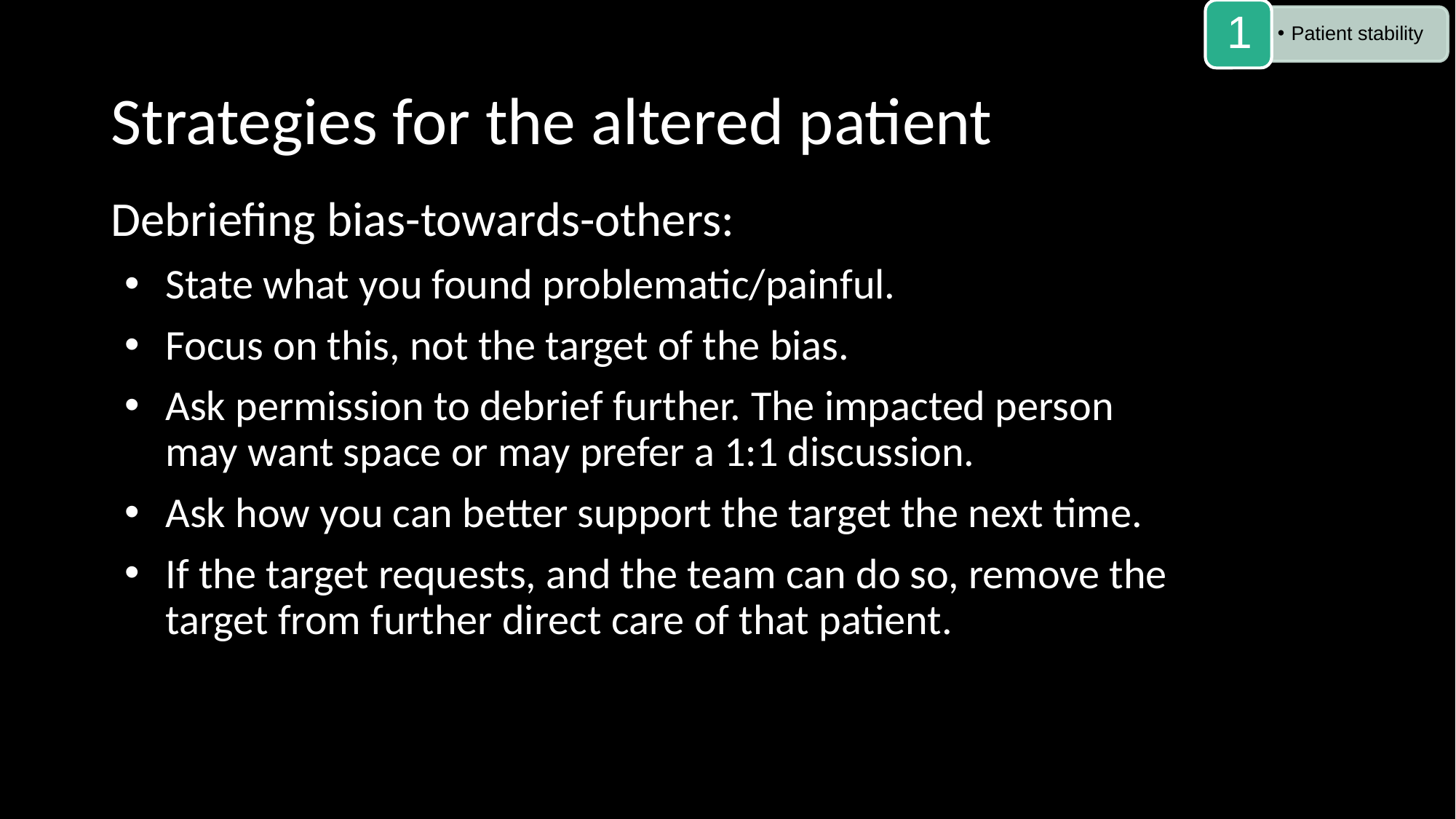

# Strategies for the altered patient
Debriefing bias-towards-others:
State what you found problematic/painful.
Focus on this, not the target of the bias.
Ask permission to debrief further. The impacted person may want space or may prefer a 1:1 discussion.
Ask how you can better support the target the next time.
If the target requests, and the team can do so, remove the target from further direct care of that patient.

## Slide 14
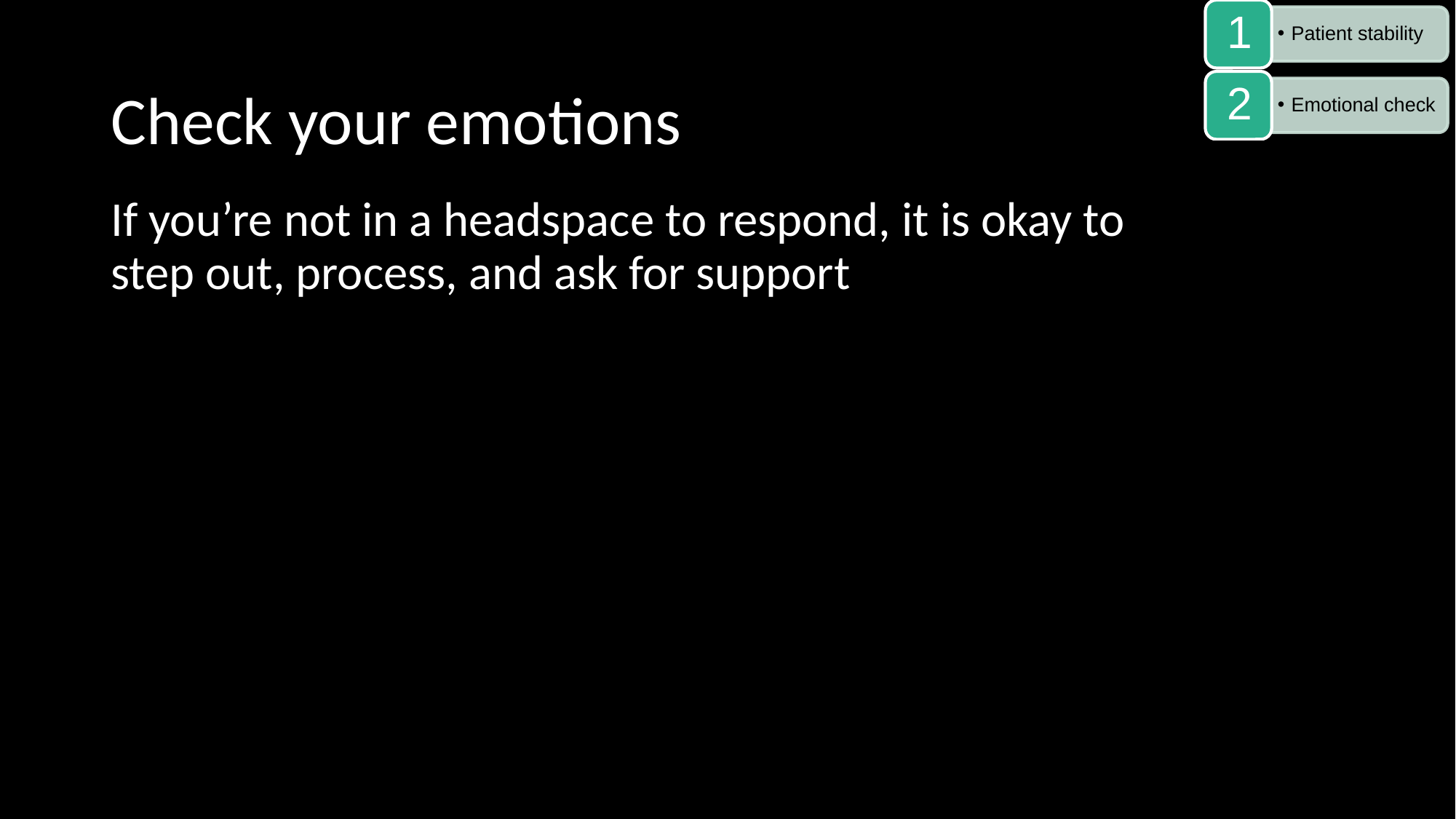

# Check your emotions
If you’re not in a headspace to respond, it is okay to step out, process, and ask for support

## Slide 15
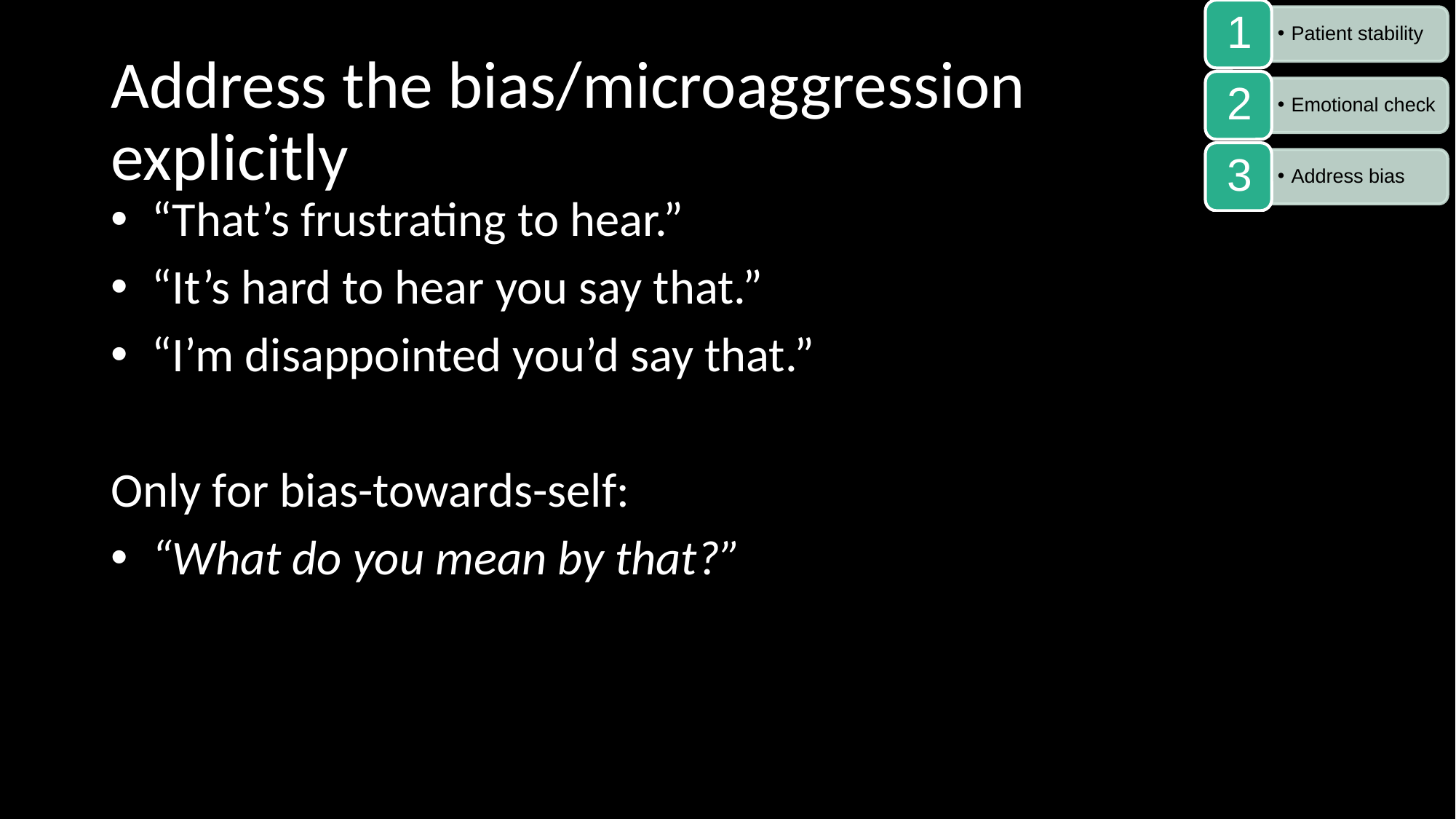

# Address the bias/microaggression explicitly
“That’s frustrating to hear.”
“It’s hard to hear you say that.”
“I’m disappointed you’d say that.”
Only for bias-towards-self:
“What do you mean by that?”

## Slide 16
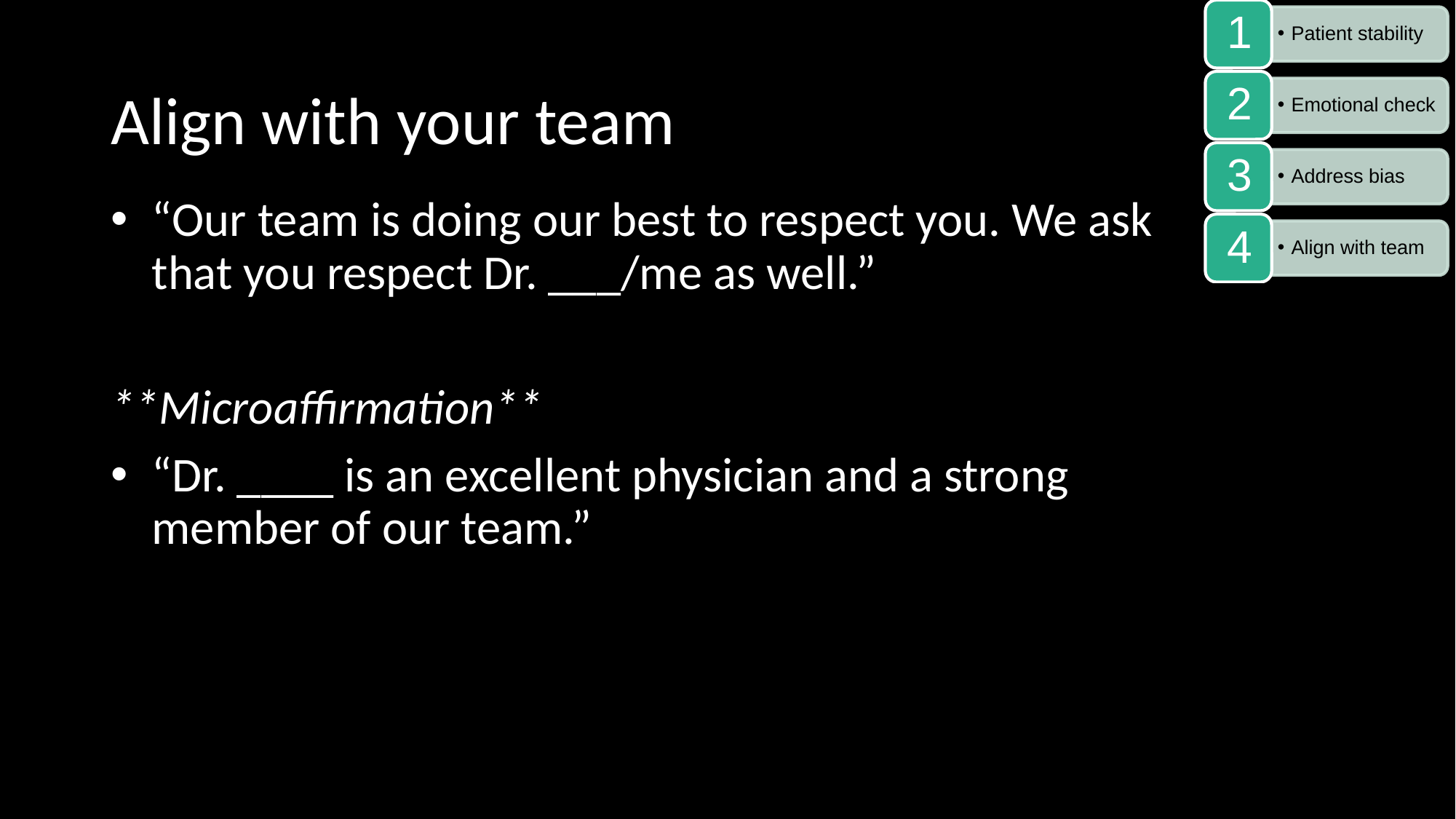

# Align with your team
“Our team is doing our best to respect you. We ask that you respect Dr. ___/me as well.”
**Microaffirmation**
“Dr. ____ is an excellent physician and a strong member of our team.”

## Slide 17
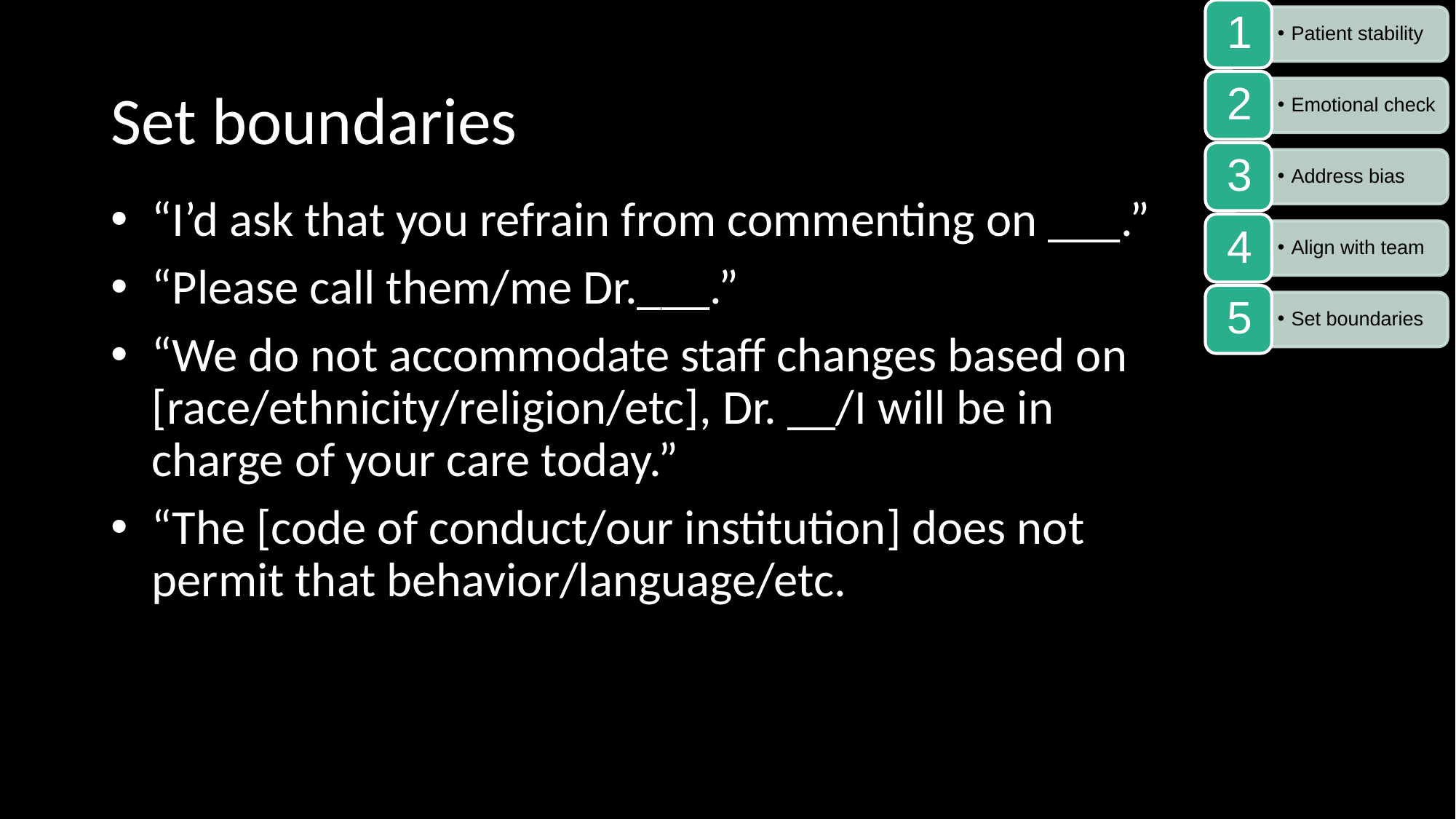

# Set boundaries
“I’d ask that you refrain from commenting on ___.”
“Please call them/me Dr.___.”
“We do not accommodate staff changes based on [race/ethnicity/religion/etc], Dr. __/I will be in charge of your care today.”
“The [code of conduct/our institution] does not permit that behavior/language/etc.

## Slide 18
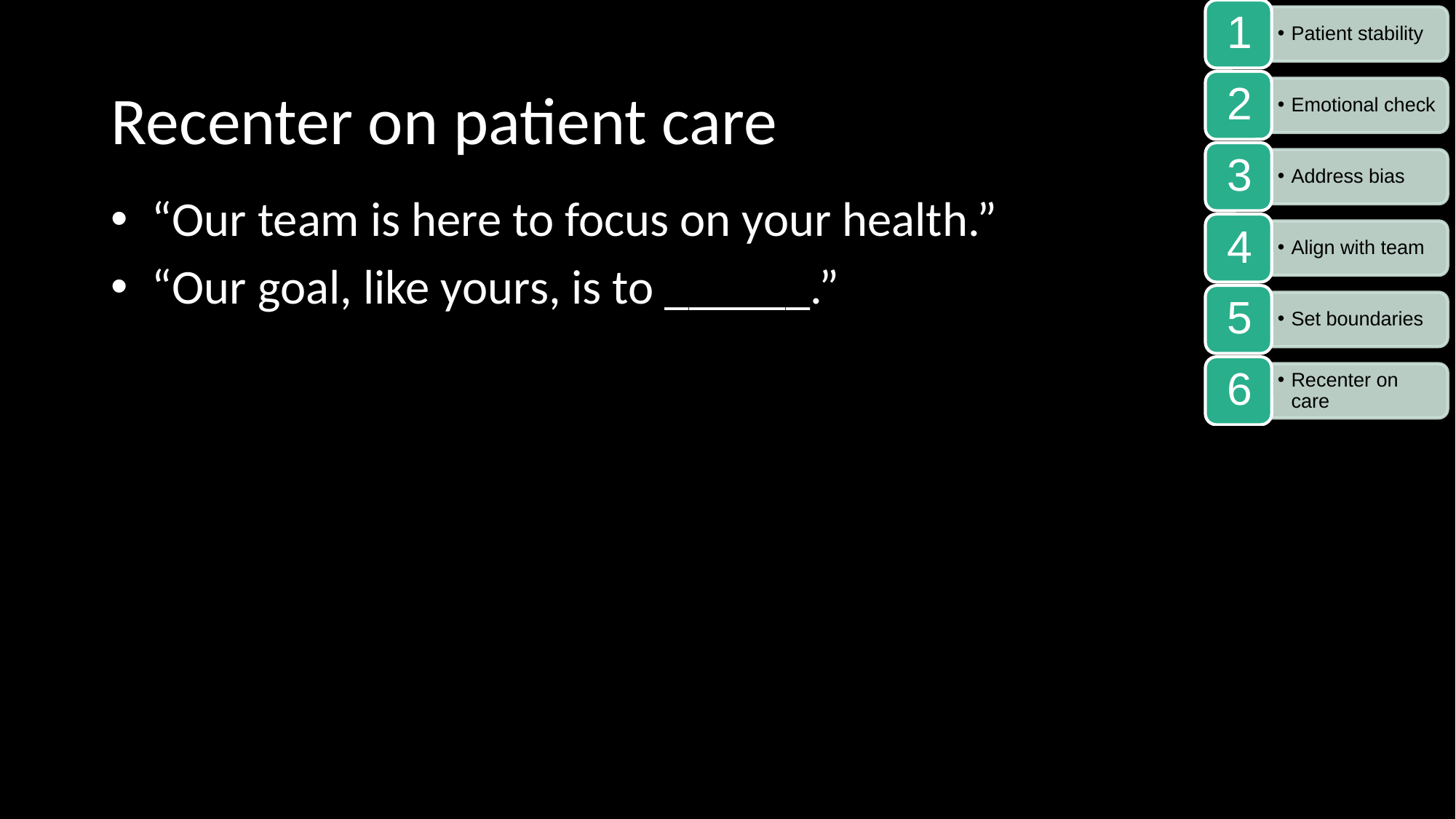

# Recenter on patient care
“Our team is here to focus on your health.”
“Our goal, like yours, is to ______.”

## Slide 19
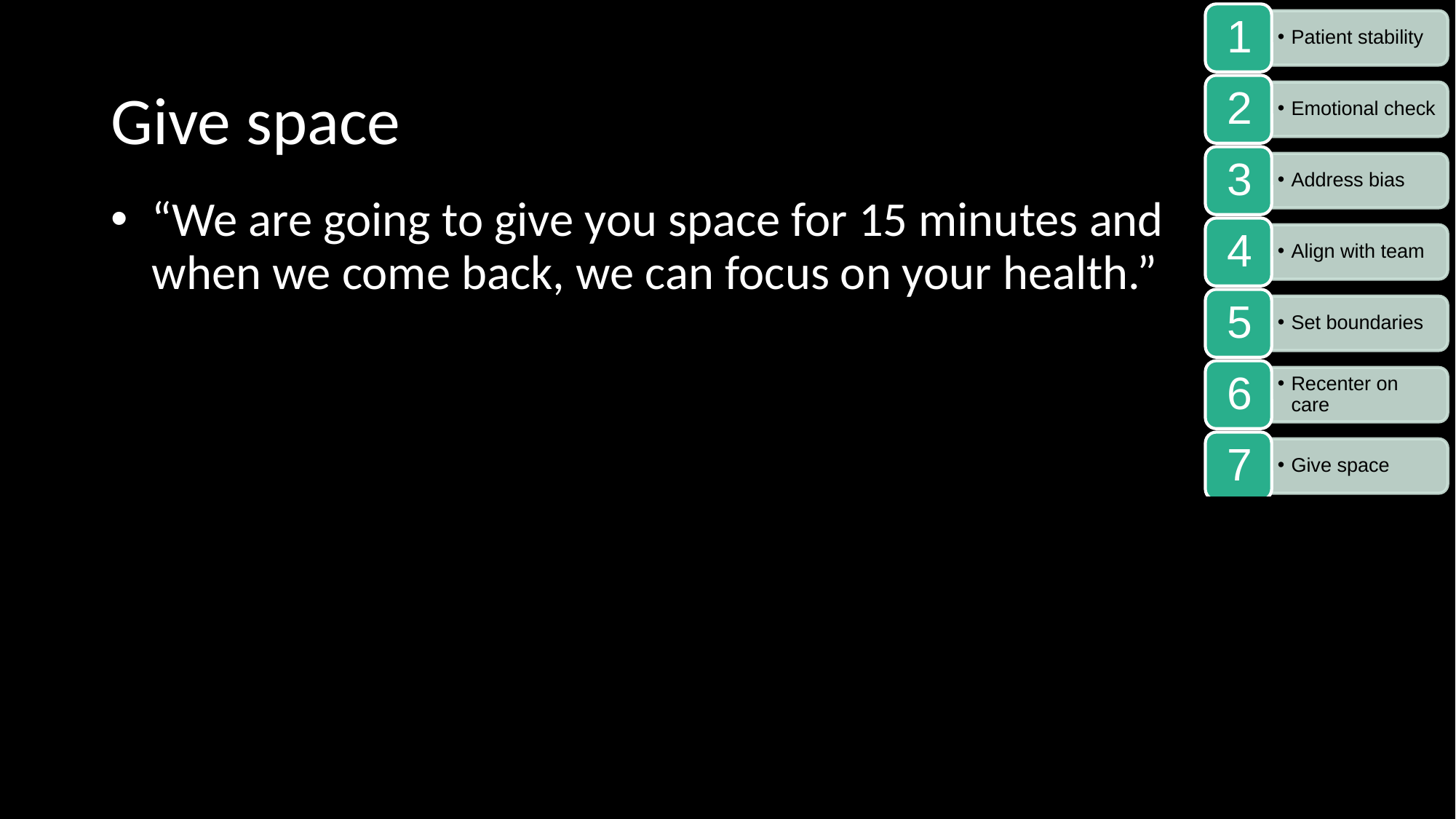

# Give space
“We are going to give you space for 15 minutes and when we come back, we can focus on your health.”

## Slide 20
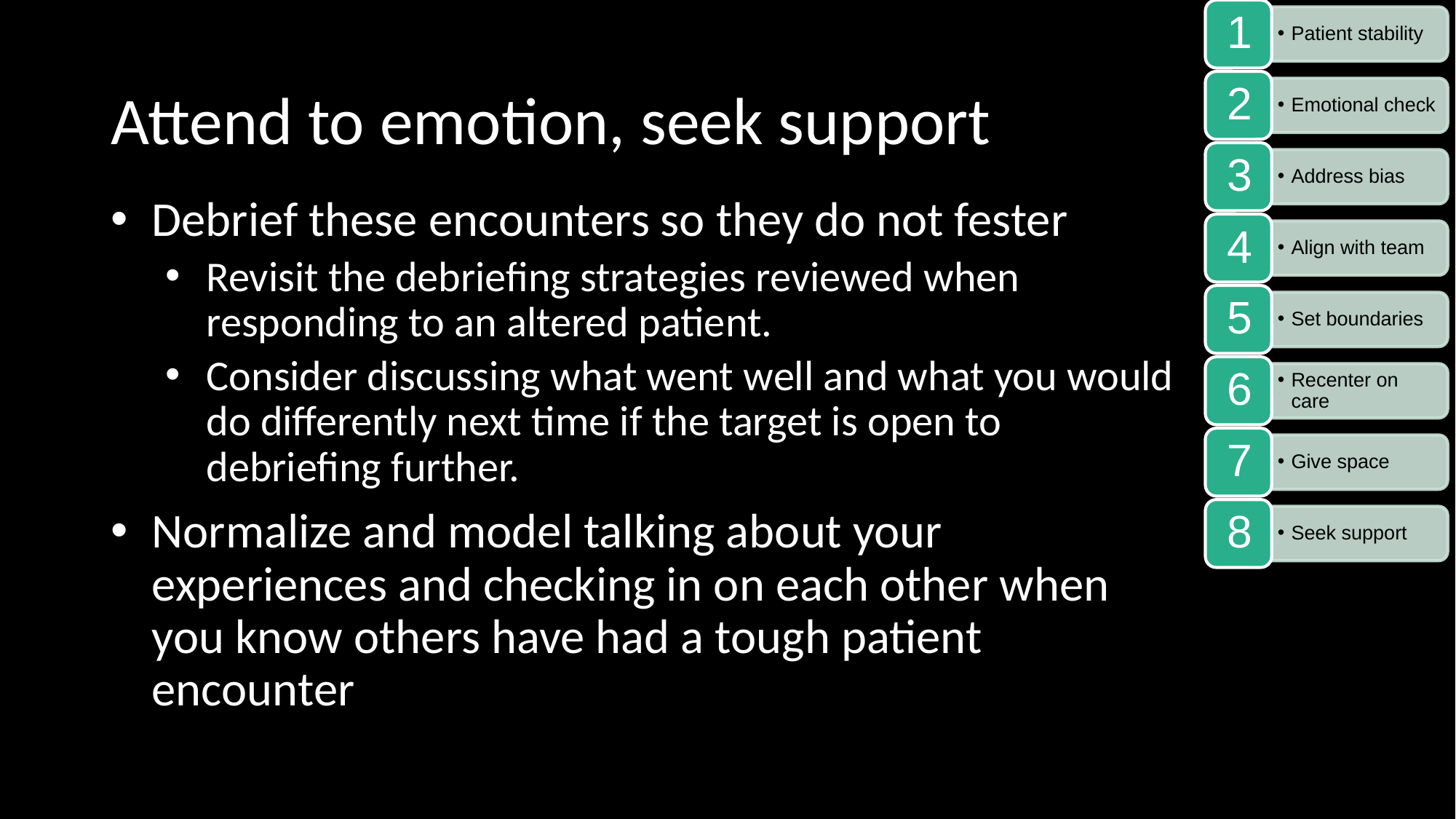

# Attend to emotion, seek support
Debrief these encounters so they do not fester
Revisit the debriefing strategies reviewed when responding to an altered patient.
Consider discussing what went well and what you would do differently next time if the target is open to debriefing further.
Normalize and model talking about your experiences and checking in on each other when you know others have had a tough patient encounter

## Slide 21
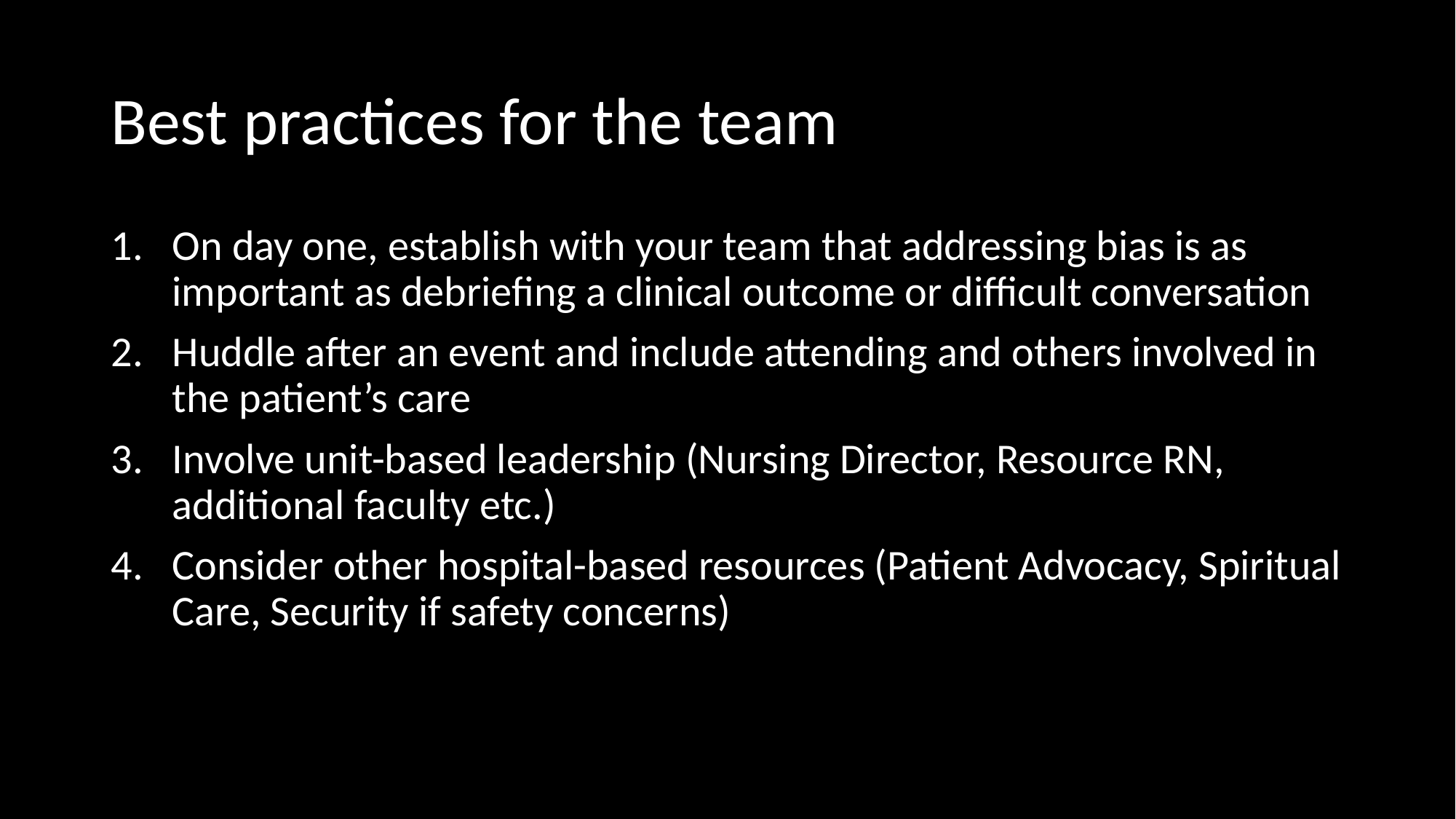

# Best practices for the team
On day one, establish with your team that addressing bias is as important as debriefing a clinical outcome or difficult conversation
Huddle after an event and include attending and others involved in the patient’s care
Involve unit-based leadership (Nursing Director, Resource RN, additional faculty etc.)
Consider other hospital-based resources (Patient Advocacy, Spiritual Care, Security if safety concerns)

## Slide 22
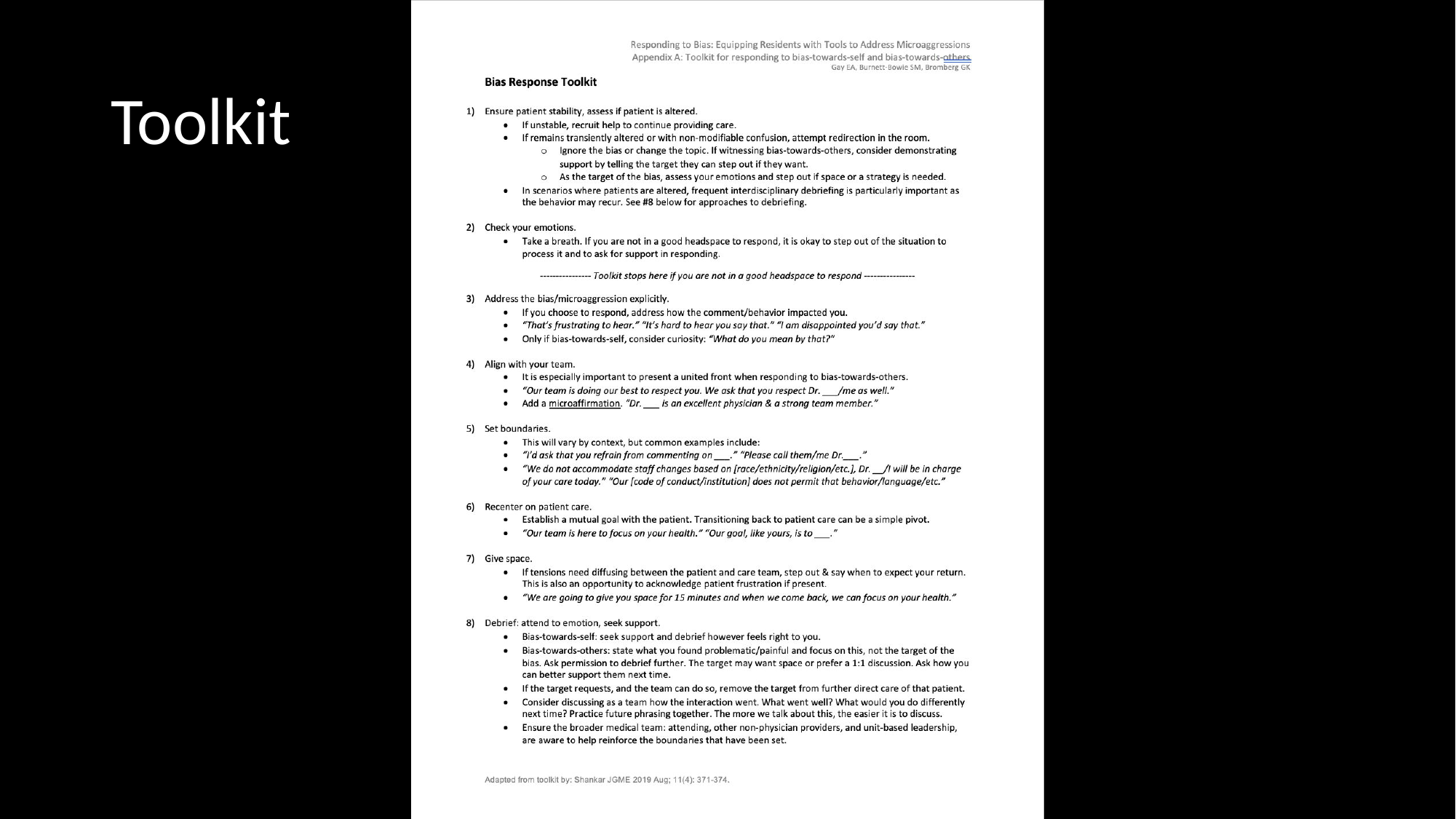

# Toolkit

## Slide 23
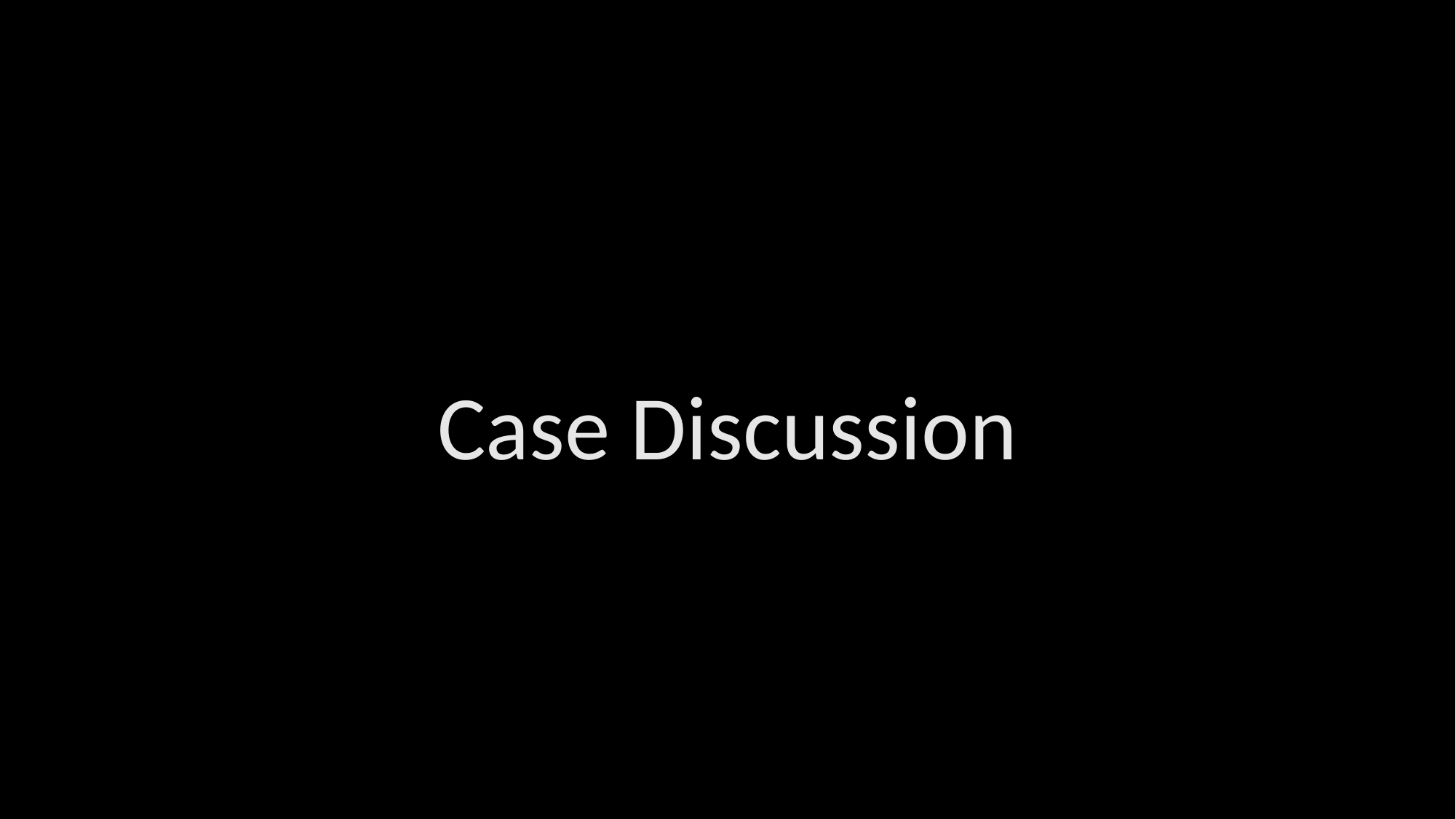

# Case Discussion

## Slide 24
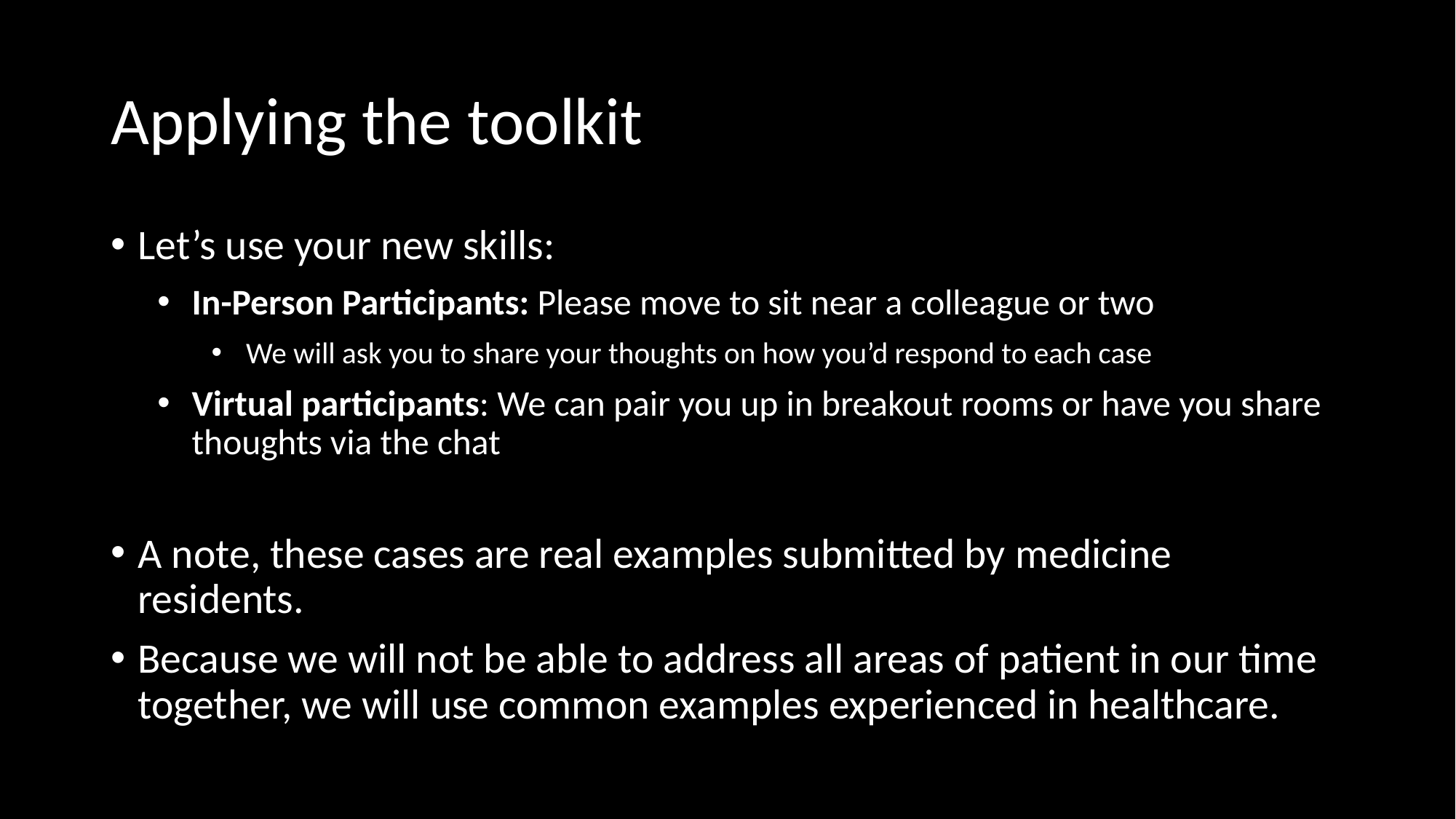

# Applying the toolkit
Let’s use your new skills:
In-Person Participants: Please move to sit near a colleague or two
We will ask you to share your thoughts on how you’d respond to each case
Virtual participants: We can pair you up in breakout rooms or have you share thoughts via the chat
A note, these cases are real examples submitted by medicine residents.
Because we will not be able to address all areas of patient in our time together, we will use common examples experienced in healthcare.

## Slide 25
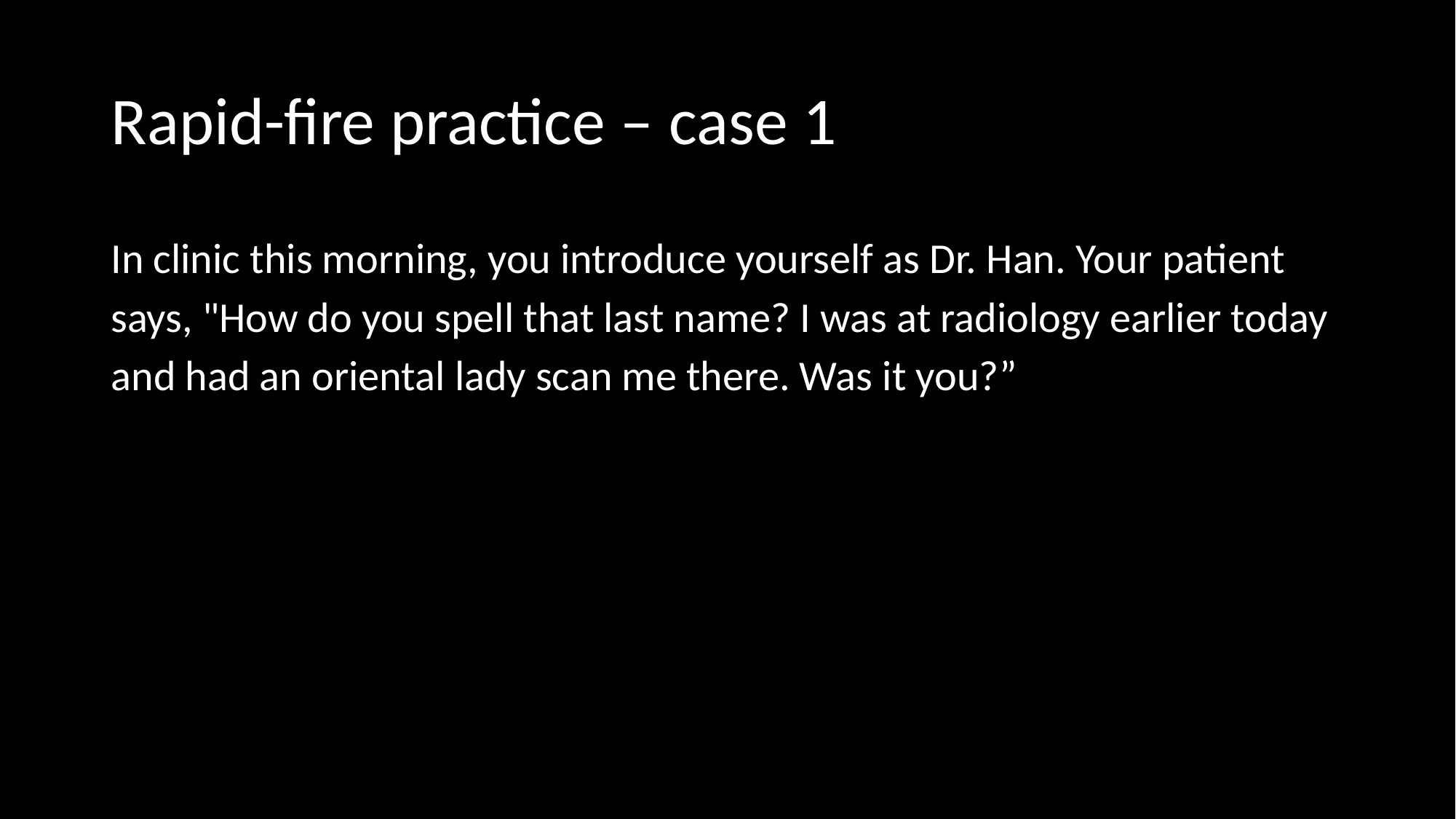

# Rapid-fire practice – case 1
In clinic this morning, you introduce yourself as Dr. Han. Your patient says, "How do you spell that last name? I was at radiology earlier today and had an oriental lady scan me there. Was it you?”

## Slide 26
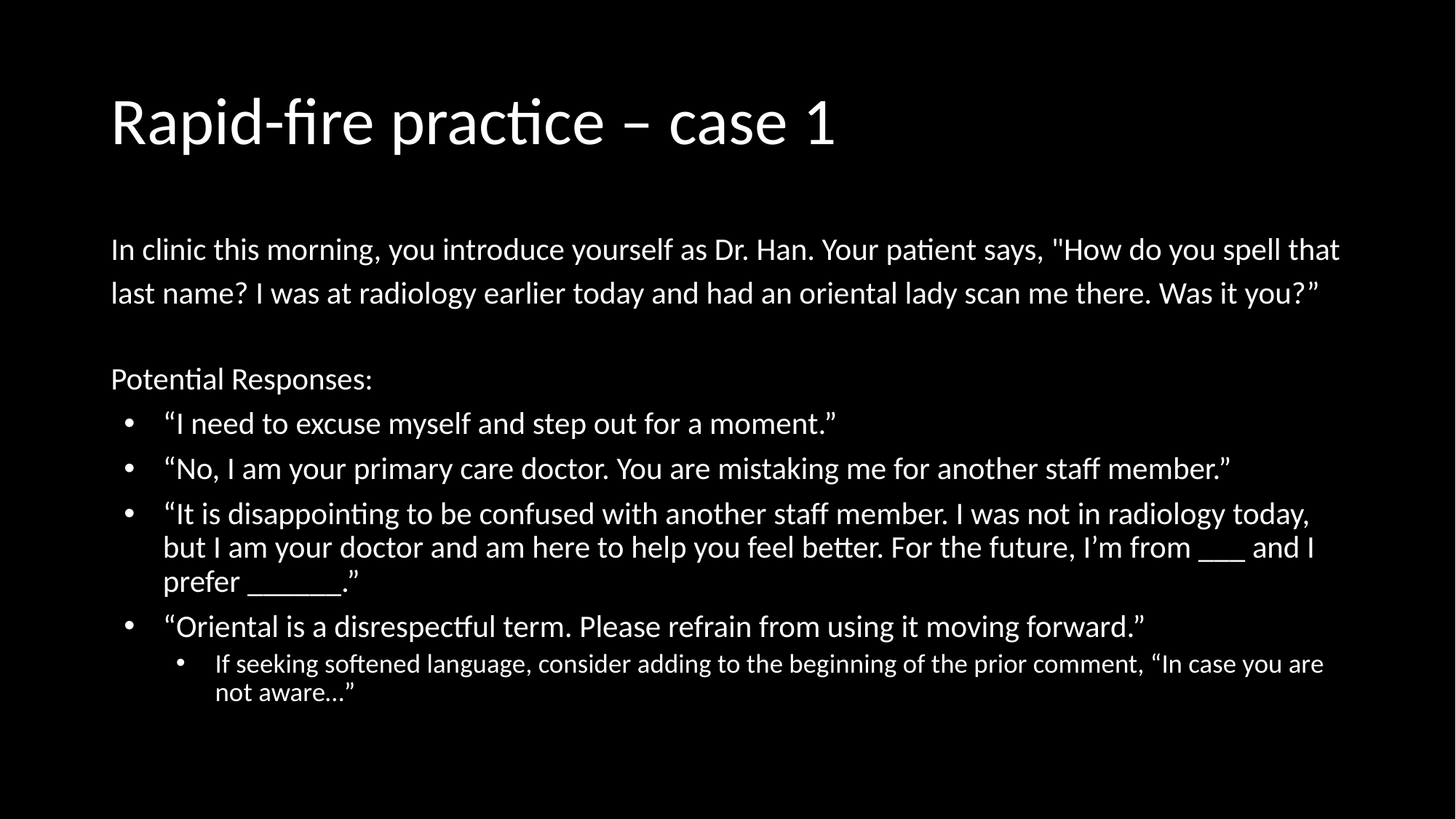

# Rapid-fire practice – case 1
In clinic this morning, you introduce yourself as Dr. Han. Your patient says, "How do you spell that last name? I was at radiology earlier today and had an oriental lady scan me there. Was it you?”
Potential Responses:
“I need to excuse myself and step out for a moment.”
“No, I am your primary care doctor. You are mistaking me for another staff member.”
“It is disappointing to be confused with another staff member. I was not in radiology today, but I am your doctor and am here to help you feel better. For the future, I’m from ___ and I prefer ______.”
“Oriental is a disrespectful term. Please refrain from using it moving forward.”
If seeking softened language, consider adding to the beginning of the prior comment, “In case you are not aware…”

## Slide 27
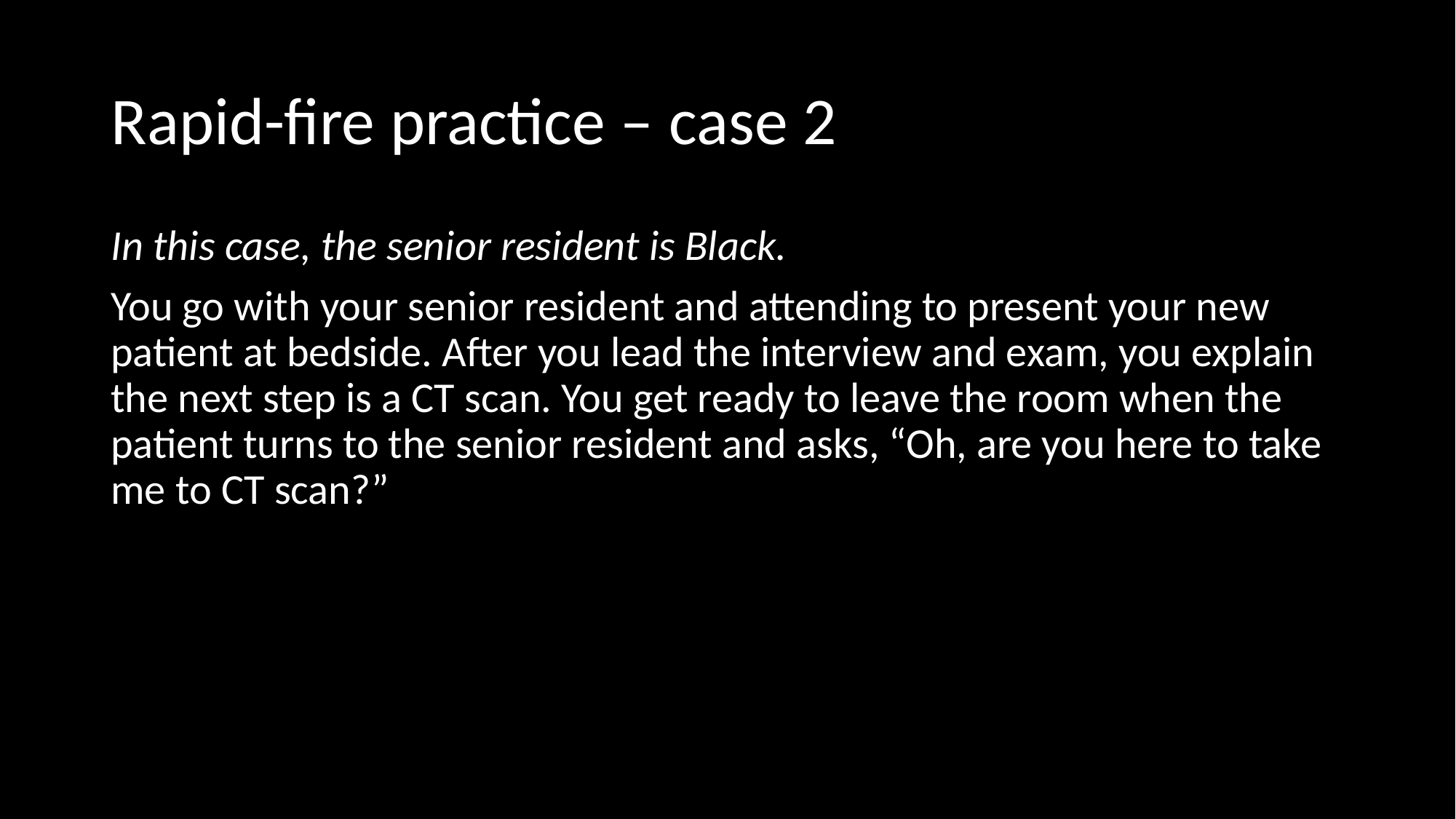

# Rapid-fire practice – case 2
In this case, the senior resident is Black.
You go with your senior resident and attending to present your new patient at bedside. After you lead the interview and exam, you explain the next step is a CT scan. You get ready to leave the room when the patient turns to the senior resident and asks, “Oh, are you here to take me to CT scan?”

## Slide 28
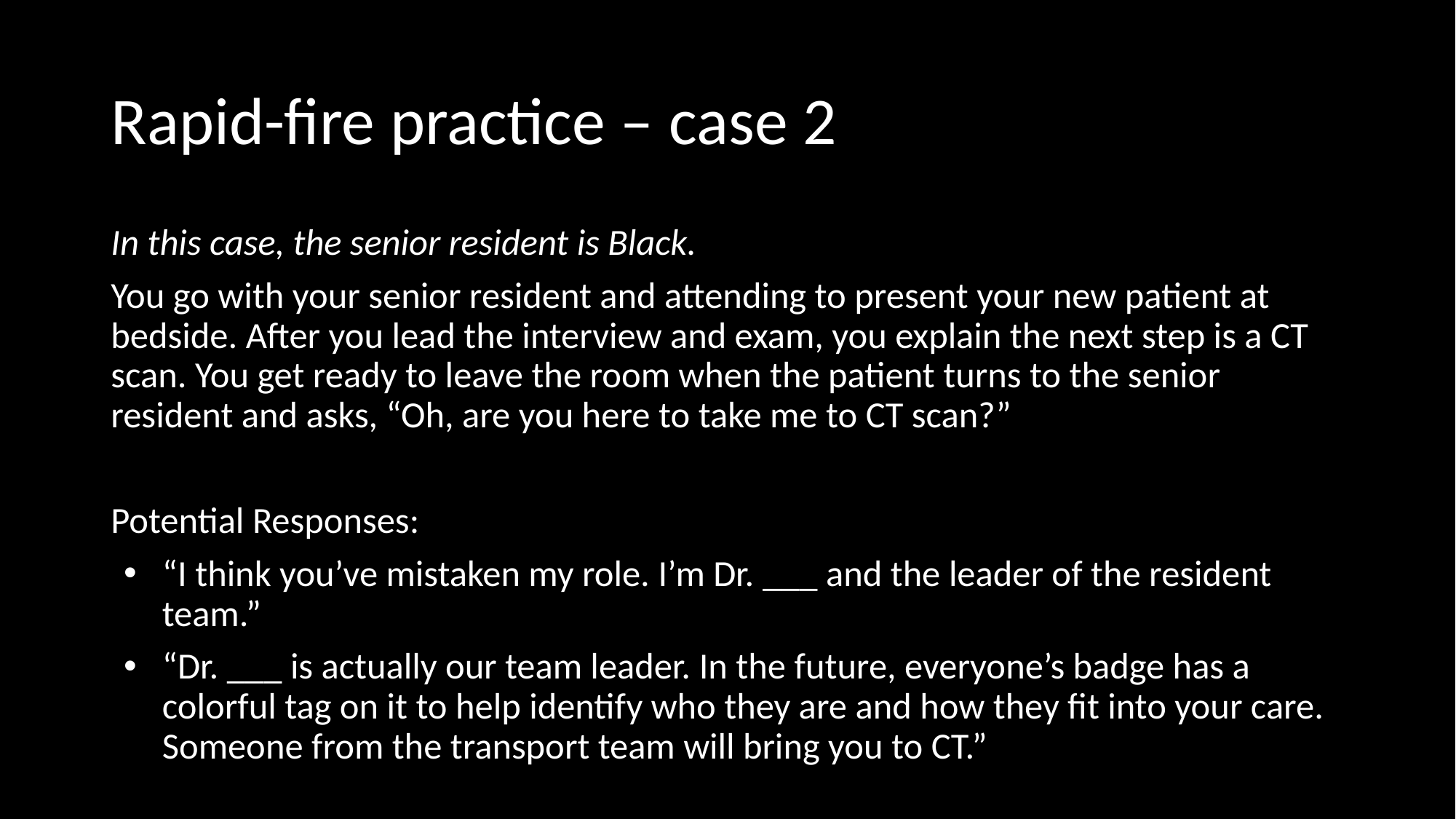

# Rapid-fire practice – case 2
In this case, the senior resident is Black.
You go with your senior resident and attending to present your new patient at bedside. After you lead the interview and exam, you explain the next step is a CT scan. You get ready to leave the room when the patient turns to the senior resident and asks, “Oh, are you here to take me to CT scan?”
Potential Responses:
“I think you’ve mistaken my role. I’m Dr. ___ and the leader of the resident team.”
“Dr. ___ is actually our team leader. In the future, everyone’s badge has a colorful tag on it to help identify who they are and how they fit into your care. Someone from the transport team will bring you to CT.”

## Slide 29
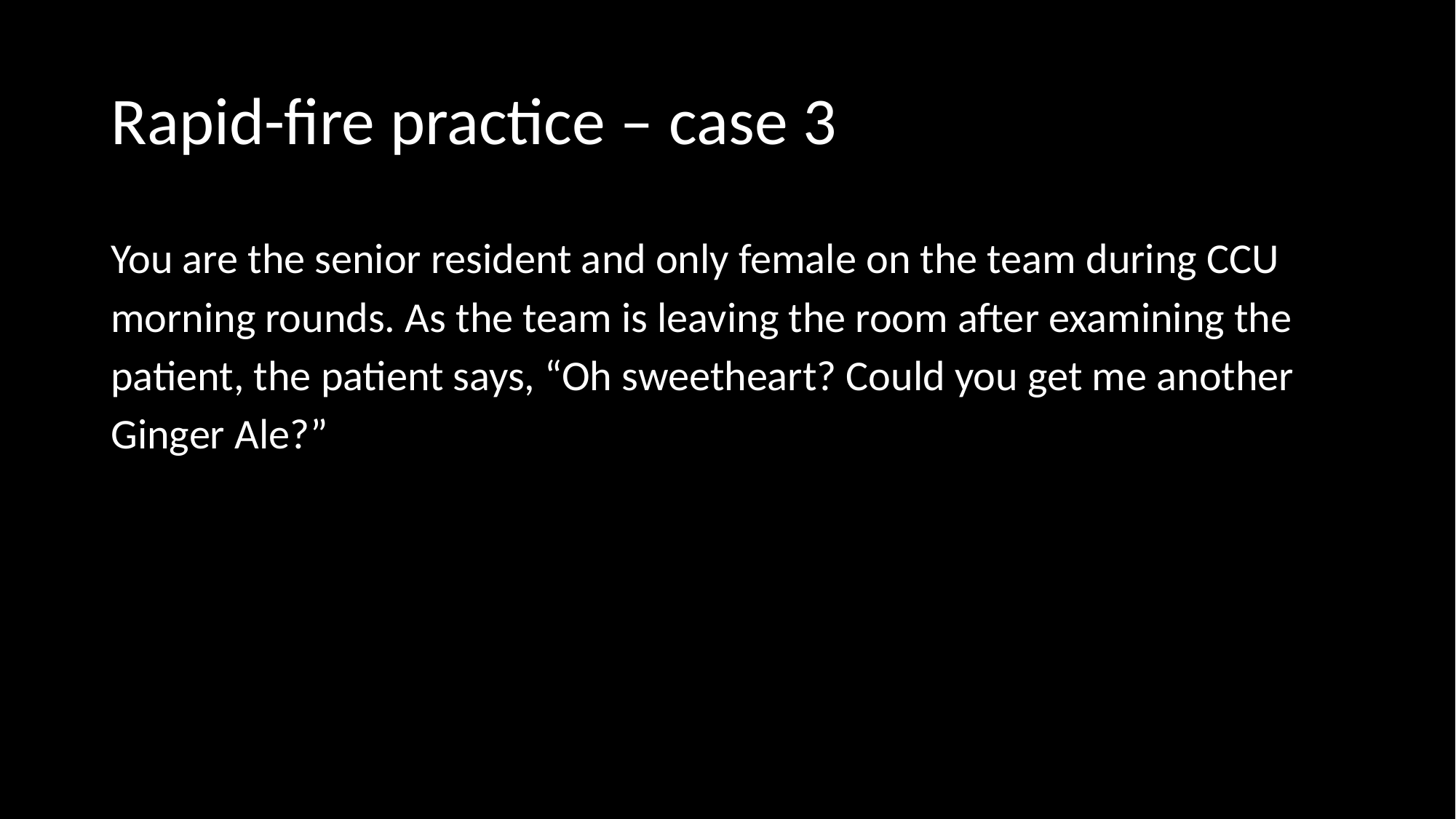

# Rapid-fire practice – case 3
You are the senior resident and only female on the team during CCU morning rounds. As the team is leaving the room after examining the patient, the patient says, “Oh sweetheart? Could you get me another Ginger Ale?”

## Slide 30
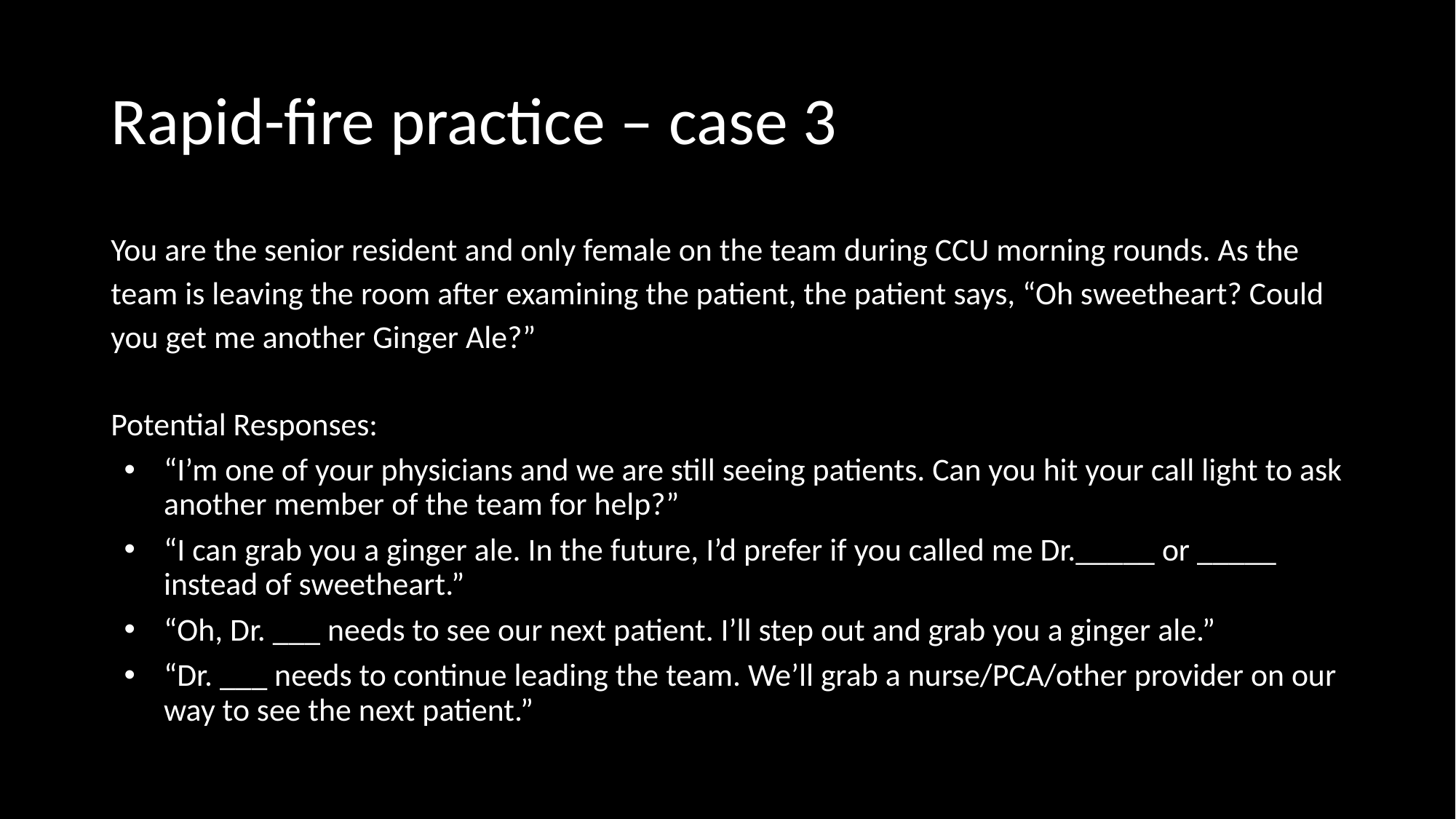

# Rapid-fire practice – case 3
You are the senior resident and only female on the team during CCU morning rounds. As the team is leaving the room after examining the patient, the patient says, “Oh sweetheart? Could you get me another Ginger Ale?”
Potential Responses:
“I’m one of your physicians and we are still seeing patients. Can you hit your call light to ask another member of the team for help?”
“I can grab you a ginger ale. In the future, I’d prefer if you called me Dr._____ or _____ instead of sweetheart.”
“Oh, Dr. ___ needs to see our next patient. I’ll step out and grab you a ginger ale.”
“Dr. ___ needs to continue leading the team. We’ll grab a nurse/PCA/other provider on our way to see the next patient.”

## Slide 31
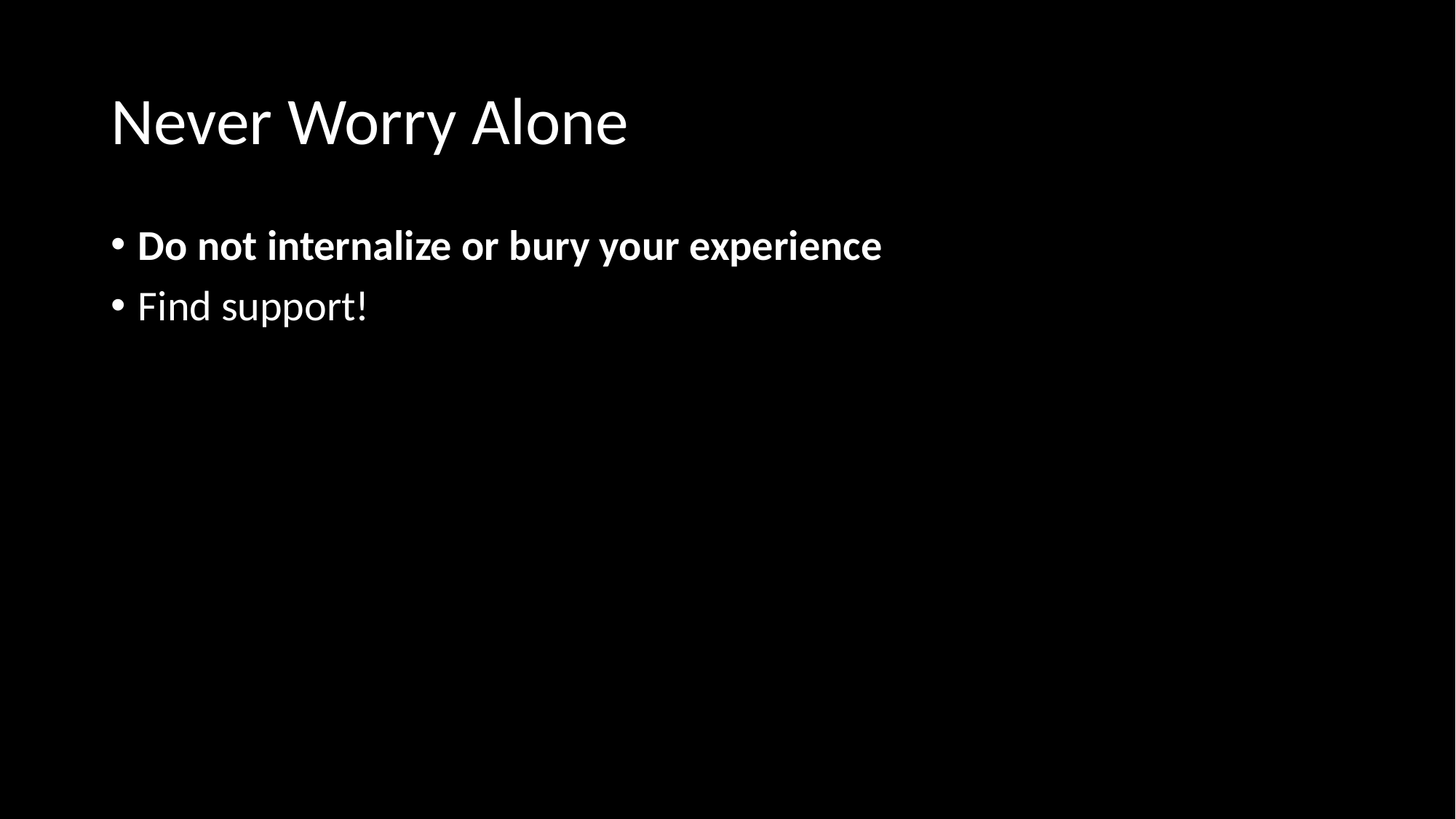

# Never Worry Alone
Do not internalize or bury your experience
Find support!

## Slide 32
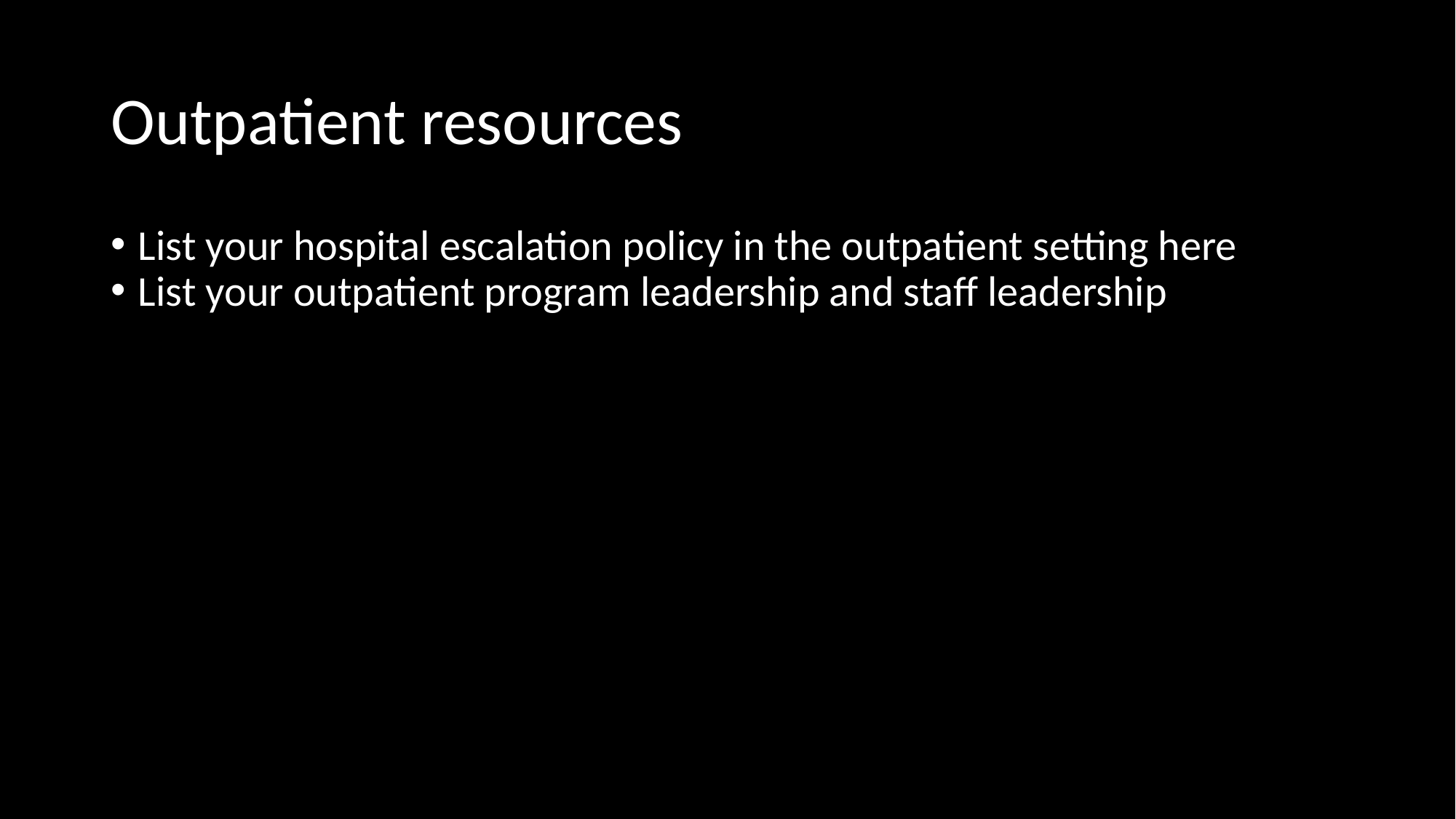

# Outpatient resources
List your hospital escalation policy in the outpatient setting here
List your outpatient program leadership and staff leadership

## Slide 33
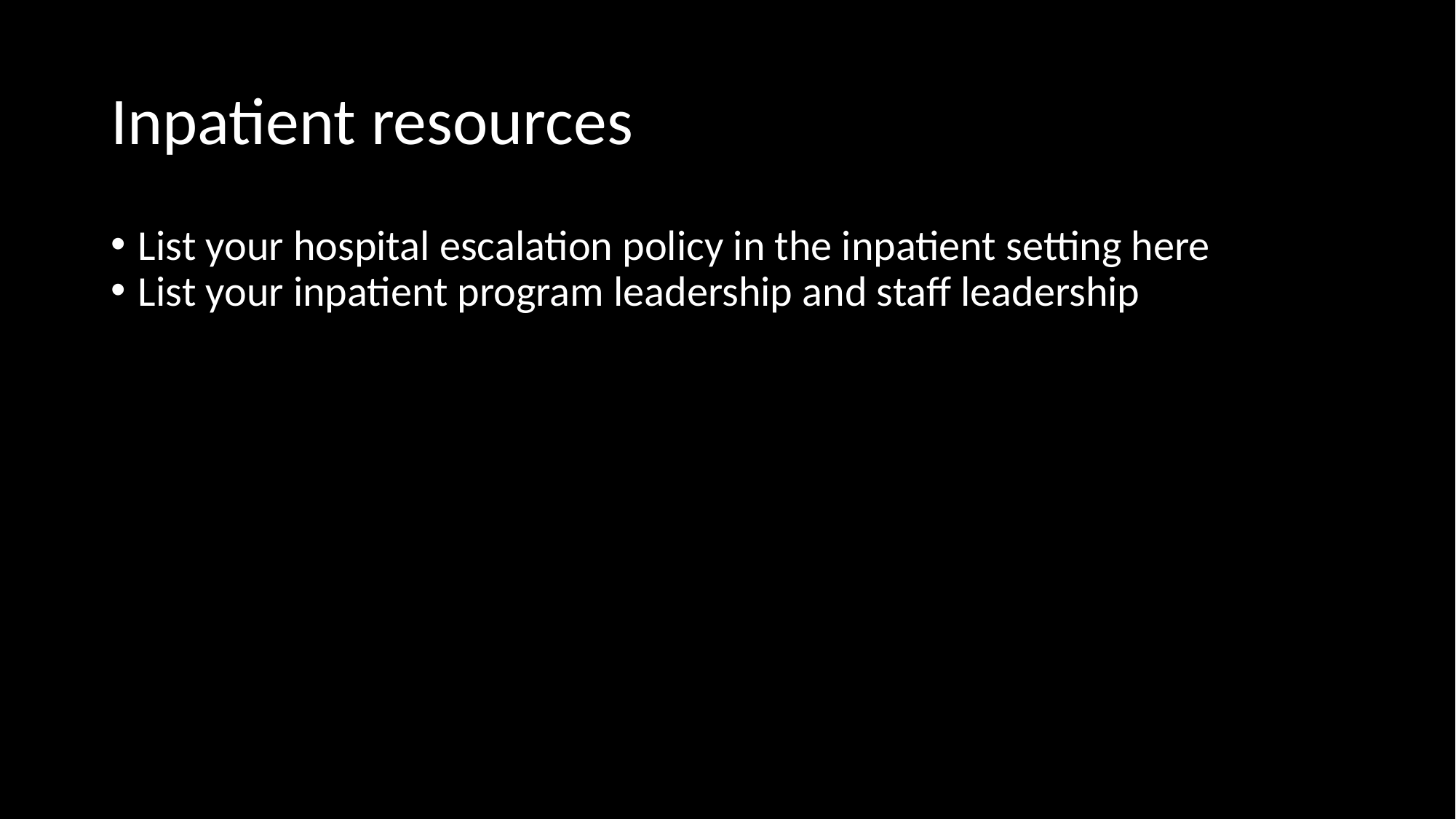

# Inpatient resources
List your hospital escalation policy in the inpatient setting here
List your inpatient program leadership and staff leadership

## Slide 34
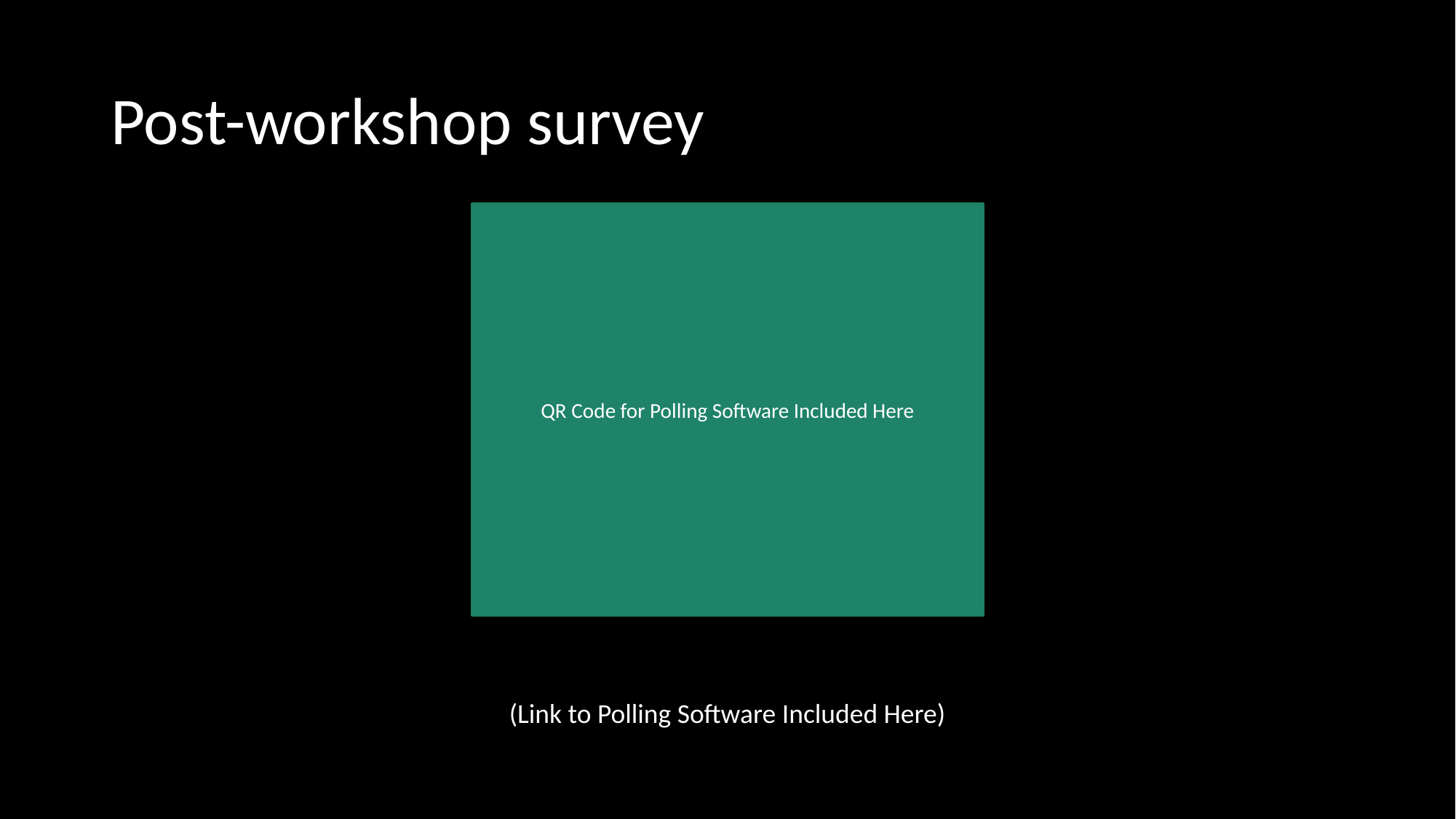

# Post-workshop survey
QR Code for Polling Software Included Here
(Link to Polling Software Included Here)

## Slide 35
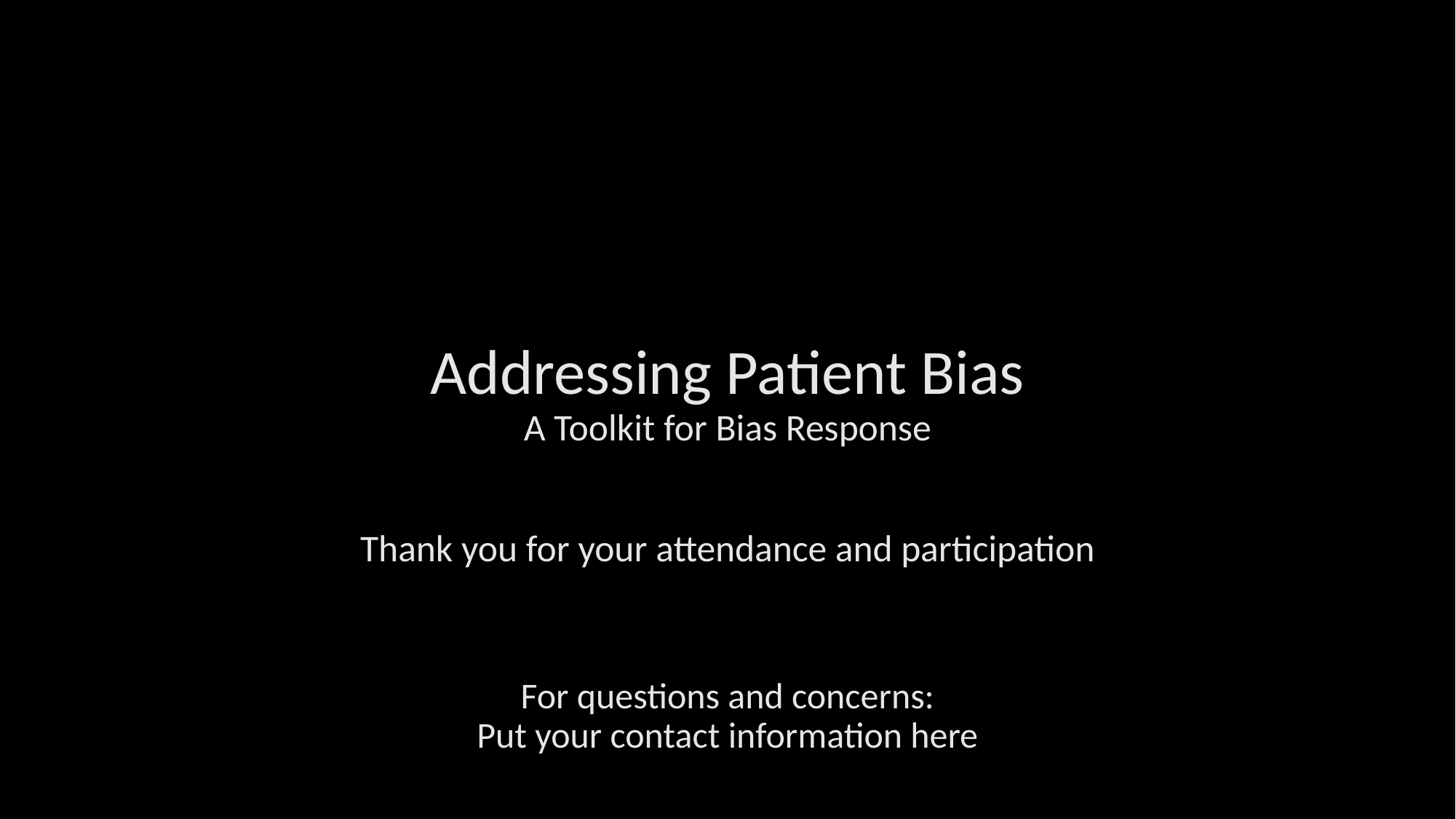

# Addressing Patient Bias A Toolkit for Bias Response Thank you for your attendance and participation
For questions and concerns:
Put your contact information here
