# Supplementary material for: Responding to Bias: Equipping Residents With Tools to Address Microaggressions
Source: MedEdPORTAL. 2024 Aug 6;20:11424. doi: 10.15766/mep_2374-8265.11424 (PMC11300577; doi:10.15766/mep_2374-8265.11424)
Supplement: Supplementary file 1 — Bias Response Toolkit.docxBias Response Workshop.pptxFacilitator Guide.docxPre- and Postworkshop Survey Questions.docx [file mep_2374-8265.11424-s001.zip › A. Bias Response Toolkit.docx]

**A. Bias Response Toolkit**

1. Ensure patient stability, assess if patient is altered.

- If unstable, recruit help to continue providing care.
- If remains transiently altered or with non-modifiable confusion, attempt redirection in the room.
  - Ignore the bias or change the topic. If witnessing bias-towards-others, consider demonstrating support by telling the target they can step out if they want.
  - As the target of the bias, assess your emotions and step out if space or a strategy is needed.
- In scenarios where patients are altered, frequent interdisciplinary debriefing is particularly important as the behavior may recur. See #8 below for approaches to debriefing.

1. Check your emotions.

- Take a breath. If you are not in a good headspace to respond, it is okay to step out of the situation to process it and to ask for support in responding.

*---------------- Toolkit stops here if you are not in a good headspace to respond ----------------*

1. Address the bias/microaggression explicitly.

- If you choose to respond, address how the comment/behavior impacted you.
- *“That’s frustrating to hear.” “It’s hard to hear you say that.” “I am disappointed you’d say that.”*
- Only if bias-towards-self, consider curiosity: *“What do you mean by that?”*

1. Align with your team.

- It is especially important to present a united front when responding to bias-towards-others.
- *“Our team is doing our best to respect you. We ask that you respect Dr. ___/me as well.”*
- Add a microaffirmation. *“Dr. ___ is an excellent physician & a strong team member.”*

1. Set boundaries.

- This will vary by context, but common examples include:
- *“I’d ask that you refrain from commenting on ___.” “Please call them/me Dr.___.”*
- *“We do not accommodate staff changes based on [race/ethnicity/religion/etc.], Dr. __/I will be in charge of your care today.” “Our [code of conduct/institution] does not permit that behavior/language/etc.”*

1. Recenter on patient care.

- Establish a mutual goal with the patient. Transitioning back to patient care can be a simple pivot.
- *“Our team is here to focus on your health.” “Our goal, like yours, is to ___.”*

1. Give space.

- If tensions need diffusing between the patient and care team, step out & say when to expect your return. This is also an opportunity to acknowledge patient frustration if present.
- *“We are going to give you space for 15 minutes and when we come back, we can focus on your health.”*

1. Debrief: attend to emotion, seek support.

- Bias-towards-self: seek support and debrief however feels right to you.
- Bias-towards-others: state what you found problematic/painful and focus on this, not the target of the bias. Ask permission to debrief further. The target may want space or prefer a 1:1 discussion. Ask how you can better support them next time.
- If the target requests, and the team can do so, remove the target from further direct care of that patient.
- Consider discussing as a team how the interaction went. What went well? What would you do differently next time? Practice future phrasing together. The more we talk about this, the easier it is to discuss.
- Ensure the broader medical team: attending, other non-physician providers, and unit-based leadership, are aware to help reinforce the boundaries that have been set.
