# Supplementary material for: Responding to Bias: Equipping Residents With Tools to Address Microaggressions
Source: MedEdPORTAL. 2024 Aug 6;20:11424. doi: 10.15766/mep_2374-8265.11424 (PMC11300577; doi:10.15766/mep_2374-8265.11424)
Supplement: Supplementary file 1 — Bias Response Toolkit.docxBias Response Workshop.pptxFacilitator Guide.docxPre- and Postworkshop Survey Questions.docx [file mep_2374-8265.11424-s001.zip › D. Pre- and Postworkshop Survey Questions.docx]

**D. Workshop Survey Questions**

As described in Appendix C’s section entitled “Prepare the survey, web conferencing tools, and toolkit,” these survey questions can be added to whichever live-polling platform is selected to accompany the workshop (Appendix B). Page 1 is the pre-workshop survey and page 2 is the post-workshop survey. If using this as a physical handout, delete these first few lines before printing for participants.

**Pre-Workshop Survey Questions**

1. How comfortable do you feel responding to a patient who has demonstrated bias towards you personally (bias-towards-self)?

| 1 | 2 | 3 | 4 | 5 | 6 | 7 |
| --- | --- | --- | --- | --- | --- | --- |
|  |  |  |  |  |  |  |
| Very uncomfortable |  |  | Neither comfortable nor uncomfortable |  |  | Very comfortable |

2. How comfortable do you feel responding to a patient who has demonstrated bias towards a colleague (bias-towards-others)?

| 1 | 2 | 3 | 4 | 5 | 6 | 7 |
| --- | --- | --- | --- | --- | --- | --- |
|  |  |  |  |  |  |  |
| Very uncomfortable |  |  | Neither comfortable nor uncomfortable |  |  | Very comfortable |

**Post-Workshop Survey Questions**

1. How comfortable do you feel responding to a patient who has demonstrated bias towards you personally (bias-towards-self)?

| 1 | 2 | 3 | 4 | 5 | 6 | 7 |
| --- | --- | --- | --- | --- | --- | --- |
|  |  |  |  |  |  |  |
| Very uncomfortable |  |  | Neither comfortable nor uncomfortable |  |  | Very comfortable |

2. How comfortable do you feel responding to a patient who has demonstrated bias towards a colleague (bias-towards-others)?

| 1 | 2 | 3 | 4 | 5 | 6 | 7 |
| --- | --- | --- | --- | --- | --- | --- |
|  |  |  |  |  |  |  |
| Very uncomfortable |  |  | Neither comfortable nor uncomfortable |  |  | Very comfortable |

3. My gender identity is:

| Man | Woman | Nonbinary or otherwise gender expansive | Other | Prefer not to say |
| --- | --- | --- | --- | --- |

4. My role is:

| Intern | Junior Resident | Senior Resident | Medical Student | Fellow | Attending | Other |
| --- | --- | --- | --- | --- | --- | --- |

5. Do you have feedback on this session? Did we address the bias you've experienced?
