# Supplementary material for: Responding to Bias: Equipping Residents With Tools to Address Microaggressions
Source: MedEdPORTAL. 2024 Aug 6;20:11424. doi: 10.15766/mep_2374-8265.11424 (PMC11300577; doi:10.15766/mep_2374-8265.11424)
Supplement: Supplementary file 1 — Bias Response Toolkit.docxBias Response Workshop.pptxFacilitator Guide.docxPre- and Postworkshop Survey Questions.docx [file mep_2374-8265.11424-s001.zip › C. Facilitator Guide.docx]

**C. Facilitator Guide (45 Minute Session)**

This guide provides facilitators a stepwise guide to preparing for and leading the workshop on addressing patient bias.

| Activity | Time | Instructions |
| --- | --- | --- |
| Prepare the PowerPoint | In advance | PowerPoint slides that require personalization:  Slide 1: Presenter names, titles, and dates. Insert polling software QR code/link for pre-workshop survey.  Slide 4: Insert polling software QR code/link.  Slide 8: Insert polling software QR code/link for word cloud responses.  Slide 21: Edit unit and hospital-based resources as applicable.  Slide 31: Insert hospital escalation policy. If there is a patient code of conduct, add it as well.  Slide 32: If applicable, list resources and policies specific to the outpatient setting. Name outpatient leadership involved in navigating patient bias.  Slide 33: If applicable, list resources and policies specific to the inpatient setting. Name inpatient leadership involved in navigating patient bias. If there is not a discrete escalation pathway for these two settings, integrate escalation resources into one slide.  Slide 34: Insert polling software QR code/link for post workshop survey.  Slide 35: Insert contact information for follow-up questions and concerns.  PowerPoint slides that could be personalized:  Slide 7: Insert local data on prevalence of bias if available. If none are available, consider including additional questions in the pre-workshop survey to better understand local context for future presentations. If not and you are teaching to a resident audience, consider changing the header of the slide to “And a problem for residents specifically…” Use this slide to paint a picture of prevalence data in a similar population.  Slide 21: While we have listed the main patient-facing resources at our institution, this list can be updated to include the names of these respective teams at your institution.  Slides 25-30: While we believe these cases to be widely applicable to all residents, consider editing cases to be relevant to the workshop participants and their usual clinical scope. Consider collecting deidentified personal experiences from participants, with permission, to use for future presentations. |
| Prepare the survey, web conferencing tools, and toolkit | In advance | Polling software:   - Load the live polling software with pre- and post-survey questions from Appendix D. Separate pre- and post- questions by a word cloud activity with the prompt “What names other than 'doctor' have patients called you?” - If not integrated into PowerPoint, the polling software should be loaded on the presenter computer prior to the session to allow for mid-presentation screen sharing of the word cloud results.   Web conference software:   - Prepare the web conference software (e.g. Zoom, Microsoft Teams, etc.). Start the conference with all online participants in one room, though prepare to create breakout rooms of 2-3 participants during the small group segments of the session. - Share your screen with the conference software so all participants can see the slides and word cloud.   Toolkit dissemination:   - Either print the toolkit to distribute in person or send via email pre- or post-workshop. |
| Prepare the room | In advance | The room must have a projector/screen with the ability to display slides from PowerPoint and a word cloud from polling software, as well as web conferencing capabilities if the session is presented in a hybrid format |
| Didactic presentation: introduction | 5 minutes | Slide 1: Introduction slide   - Introduce the workshop as “Addressing Patient Bias: A Toolkit for Bias Response.” Presenters can introduce themselves and acknowledge their identities/privileges as appropriate. Despite the limitations of everyone’s perspective, the shared goal is to create a safe space to learn to discuss and respond to bias from patients when it occurs. Share that the hope is to learn to support each other in these experiences and cultivate an institutional culture of responding to these events. - Before moving on to the next slide, ask that participants use the QR code to answer pre-workshop questions—the purpose of the survey is to ensure the workshop is successful in building attendee confidence in responding to patient bias. Encourage honest feedback so the workshop can be improved. If polling software is anonymous, share that with participants.   Slide 2: Roadmap   - Briefly discuss the agenda for the workshop: 1) Discuss the background on patient bias, both nationally and locally. 2) Warm up exercise with an anonymous word cloud. 3) Present a toolkit for a framework and example language to responding to patient bias-towards-self and bias-towards-others. 4) Practice this toolkit with real cases in small groups and share with the large group.   Slide 3: Learning Objectives   - Briefly discuss the objectives for the workshop: 1) Create a safe environment to share experiences and approaches to dealing with patient bias 2) Recognize examples of bias in clinical encounters 3) Develop a toolkit to respond to bias. - Establish expectations of confidentiality and respect. - Explicitly state that this conversation can be triggering for some, and that bias is not experienced equally by residents. The choice to take space or step out is respected. There is no expectation of participation. - Acknowledge these objectives must exist in a larger institutional response to bias and that these supports and resources will be shared at the end of the presentation. - Remind virtual learners to use the hand raise function if unmuting to participate.   Slide 4: Survey   - Offer a final opportunity to take the pre-workshop survey. |
| Didactic presentation: define bias and its prevalence | 3 minutes | Slide 5: Definitions   - To ensure a shared language for the session, share definitions of bias, microaggression, stereotype, and discrimination with the group. Acknowledge the term microaggression is somewhat of a misnomer and does not mean the impact of microaggression on residents is ‘micro,’ but that micro evokes these acts’ common, interspersed nature.   Slide 6: National prevalence   - Share national data on the frequency of patient bias. Point out that the experience of bias is affected by clinician race/ethnicity and that physicians of color bear the brunt of these experiences.   Slide 7: Local prevalence   - If there is local data available on the frequency of patient-expressed bias, add and share it here. - If local data are not available, share that based on the surveyed intern class in a large academic, urban Internal Medicine residency, bias related to race and gender are frequently both experienced and observed by residents. The most common source of this bias was patients and their families. Residents also experienced many other forms of bias which you can see listed on the slide. |
| Audience participation: word cloud | 2 minutes | Slide 8:   - Introduce the word cloud activity. Ask participants to open the polling application and answer the question “What names other than doctor have patients called you?” - If using non-embeddable polling software, switch the screen share from PowerPoint to the polling software so the free text responses are seen in real time. Read a few of the names out loud as they come up. - After about 30 seconds, comment on some of the largest/most common text inputs. Offer that these examples of misidentification are likely a fraction of the patient-expressed bias the audience has experienced. - If time allows, ask participants what they notice about the word cloud: Are there themes in the names? What biases are unveiled here? Has anyone struggled to respond to being called one of these names? Remember to check the virtual conference chat for responses as well. - If using non-embeddable polling software, switch the screen back to PowerPoint. |
| Intervention training: the toolkit | 5 minutes | Slide 9: Transition   - Ask participants to put down their phones and to leave the polling software open for the post-workshop survey. - Mark the transition to the intervention training with the toolkit. Explicitly state this toolkit is useful both when responding to bias-towards-self and to bias-towards-others.   Slide 10: Toolkit overview   - Briefly introduce all steps of the toolkit, remembering you are about to proceed with an in-depth review of each step. Remind learners that this toolkit is designed to be applied when responding to both patient bias-towards-self and bias-towards-others.   Slides 11-20: Stepwise toolkit review   - Introduce each step of the toolkit with example bias response language.   Slide 21: Team best practices   - Establish best practices around patient-expressed bias with each team. Suggest that all teams will experience bias from a patient and that taking time to debrief and process these events is critical. The presenter can offer that, for example, at the beginning of a rotation each team can discuss each team member’s experiences with bias. Some team members might share they need time and space between the event and debrief, others might share they still struggle responding to patient-expressed bias and would like team members to respond for them, whereas some might share that they want to speak for themselves should an event occur. - Presenters should emphasize that attendings, nursing, staff and others experience patient-expressed bias as well and are valuable members to bring into the conversation. Discuss how it takes the whole medical team to establish boundaries and enforce consequences if a behavioral contract is required. - The presenter can offer patient support resources as well. Ensure this is updated to reflect your institution’s resources in advance. At our institution, patient-facing resources include patient advocacy and spiritual care. Involve security if there is a concern.   Slide 22: Toolkit summary   - Provide the toolkit in paper or electronic form to those in person and in PDF form to those on the virtual platform to use during the cases. - Remind participants the toolkit will be distributed via email. - Encourage participants to practice their favorite phrases so they roll off the tongue during a stressful situation. |
| Toolkit application: rapid fire cases | 25 minutes | Slide 23: Case transition   - Announce the transition to applying the toolkit.   Slide 24: Case transition   - Ask participants in person to move to sit near a colleague or two to form groups of 2-3 people. - Remind attendees that the cases about to be discussed are from current residents. - Explain that each small group will have a few minutes to discuss how they would respond to the case using the toolkit (they should have the toolkit either as a hard copy in front of them or in their email, while the cases will be displayed on the slides at the front of the room). - Ask all members who feel comfortable chiming in to participate in their groups’ discussions of the three cases. - Ask each group to identify a spokesperson to share the group’s discussion with the broader workshop later. - If there are at least 2 virtual participants and you have a second facilitator or collaborator who can place those participants into a breakout room of 2-3, you can offer this. If virtual participants are not able to join a breakout room and verbally participate, offer them the chat function to share their proposed solutions with each other either in dyads via direct message, or with the broader group. The hope is to make the virtual space as psychologically safe as possible. - Remind participants they can step out to collect themselves in these encounters.   Slides 25 – 30: Cases  Overall architecture for each slide:   - Read the case out loud or consider soliciting a reader from the audience. Ask participants to discuss a response using the toolkit within their small groups. Leave the case slide visible so participants can reference it during their breakouts. - Allow 3 minutes for small group resident discussion.   - Split virtual participants into break out rooms or ask them to submit responses in the chat.   - Circulate during this discussion to assess if discussion is petering off. If groups are wrapping up or have moved on to discussing other topics, pivot back to the large group discussion sooner. Check the chat for responses as well.   - If in breakout rooms, bring the virtual participants back to the main room. - Allow 5 minutes for large group, presenter-facilitated reflection.   - Bring the focus back to the front of the room. Ask the broader group to identify the bias demonstrated in the case.   - Ask groups to share their responses. If applicable, read examples from the chat. Acknowledge the tools used from the toolkit that participants are employing (naming the bias, setting a boundary, etc.).   - If participation is minimal, share potential responses that have been pre-crafted by advancing the slide forward (example on the bottom half of the slide) and ask learners if they feel they would be comfortable employing these examples.   - If time allows, each case has additional prompts for the group to deepen the discussion. For all cases, examples include “what makes this scenario challenging?” “What [demographic] assumptions did you make about those depicted in this case?” Additional examples are included in the slides. - Repeat for as many cases as time allows (likely 3 cases in 45 minutes)   Specifics for each case  Slides 25-26—Bias against Asian residents / racial bias   - This case highlights bias against Asian residents. Note that misidentification happens to providers of other races/ethnicities as well. Remind participants they can step out to collect themselves in these encounters. Consider pushing learners to specifically discuss how they would set the record straight in naming their role on the patient’s care team and in correcting inappropriate language. - How to name your role, “No, I am your primary care doctor. You are mistaking me for another staff member.” Or “It is disappointing to be confused with another staff member. I was not in radiology today, but I am your doctor and am here to help you feel better. For the future, I’m from ___ and I prefer ______.” - How to correct inappropriate language, “Oriental is a disrespectful term. Please refrain from using it moving forward.” If seeking a softer approach, consider adding, “In case you are not aware…” to the beginning of the prior comment.   Slides 27-28—Bias against Black residents / racial bias   - This case highlights racial bias. This case is an opportunity to respond to bias-towards-others and to support your peers. - How to respond to bias-towards-self, “I think you’ve mistaken my role. I’m Dr. ___ and the leader of the resident team.” - How to respond to bias-towards-others, “Dr. ___ is actually our team leader. In the future, everyone’s badge has a colorful tag on it to help identify who they are and how they fit into your care. Someone from the transport team will bring you to CT.” - Cases of responding to bias-towards-others can be fruitful opportunities to discuss how to debrief an interaction if time allows. Consider asking the group how a responding team member might do an emotional check in with the senior resident after the event. Consider asking the group what the colleague might say to the senior after the event if they froze and didn’t respond to the bias on their behalf. Alternatively, what might the senior say to the colleague if the opportunity to respond to bias-towards-others was missed?   - Language to check in with the senior resident, “I found it frustrating that the patient misidentified you as transport. How are you feeling? Do you want to discuss it further?”   - Language to check in with the senior resident if no one jumped in to respond to the bias, “I found it frustrating that the patient misidentified you as transport and I am sorry I did not jump in to support you. How can I better support you in the future?”   - Language the senior could use if no one jumped in on their behalf to respond to the bias, “I found it frustrating to be misidentified as transport by our patient. I hope in the future you feel comfortable verbally supporting me. I find xyz supportive.”   Slides 29-30—Gender Bias   - This case highlights gender bias or potentially another bias (race, ethnicity, age, etc.) depending on the target. The presenter can suggest that women experience this type of role confusion and inappropriate terms of endearment more than men. This case is an opportunity to both respond as the target to bias-towards-self, or for a peer if bias-towards-others. - How to respond to bias-towards-self, “I’m one of your physicians and we are still seeing patients. Can you hit your call light to ask another member of the team for help?” Or “I can grab you a ginger ale. In the future, I’d prefer if you called me Dr._____ or _____ instead of sweetheart.” - How to respond to bias-towards-others, “Oh, Dr. ___ needs to see our next patient. I’ll step out and grab you a ginger ale.” Or “Dr. ___ needs to continue leading the team. We’ll grab a nurse/PCA/other provider on our way to see the next patient.” |
| Institutional Supports:  resources | 3 minutes | Slide 31: Seek support   - Transition to closing resources. These experiences can be isolating, but residents should never feel that they need to process alone. Offer that presenters can stay back after the conference to debrief as needed and participants should feel empowered to leave anonymous comments on the post-workshop survey. - Emphasize that there is institutional support to respond to bias. List the hospital escalation policy and patient behavioral code of conduct, if applicable.   Slide 32: Outpatient resources   - List outpatient resources and policies around patient bias. List leaders in the outpatient arena who are committed to supporting residents in responding to bias.   Slide 33: Inpatient resources   - List inpatient resources and policies around patient-expressed bias. List leaders in the inpatient arena who are committed to supporting residents in responding to bias. |
| Closing | 2 minutes | Slide 34: Post-workshop survey   - Encourage participants to complete the post-workshop survey and leave anonymous feedback to improve the workshop for the future.   Slide 35: Closing   - Presenters thank attendees for their participation and offer contact information for concerns or questions. |
| Distribute the Toolkit | After Workshop | Resources   - Send out toolkit and escalation resources via email to participants. |
