# Supplementary material for: Responding to Bias: Equipping Residents With Tools to Address Microaggressions
Source: MedEdPORTAL. 2024 Aug 6;20:11424. doi: 10.15766/mep_2374-8265.11424 (PMC11300577; doi:10.15766/mep_2374-8265.11424)
Supplement: Supplementary file 1 — Bias Response Toolkit.docxBias Response Workshop.pptxFacilitator Guide.docxPre- and Postworkshop Survey Questions.docx [file mep_2374-8265.11424-s001.zip › _Educational Summary Report_11424.pdf]

MedEdPORTAL® The AAMC Journal of Teaching and Learning Resources

Original Publication Open Access

Responding to Bias: Equipping Residents With Tools to Address Microaggressions

Elizabeth A. Gay, MD, Sherri-Ann M. Burnett-Bowie, MD, MPH†, Gabrielle Kis Bromberg, MD*†

*Corresponding author: gbromberg@mgh.harvard.edu

†Co-senior author

Abstract

Introduction: Resident physicians frequently experience bias at work, with patients and families often being the source. Women and other trainees underrepresented in medicine are disproportionately impacted by these negative experiences, and experiencing bias contributes to resident physician burnout. Unfortunately, many resident physicians feel inadequately prepared to respond to bias. **Methods:** We developed a 45-minute, peer-led, case-based workshop that equipped trainees with tools to respond to patient-expressed bias. Our toolkit centered on resident physicians by including an assessment of the trainee's emotional well-being, a team-based response, and an embedded debrief. The toolkit provided resident physicians with possible responses to bias directed towards themselves (bias-towards-self) or bias directed towards others (bias-towards-others). Surveys were administered pre- and postworkshop to assess change in participants' comfort in responding to patient-expressed bias. **Results:** Thirty-seven residents completed both surveys. The workshop significantly increased comfort in responding to bias-towards-self ($p < .001$; 95% CI, 1.00-1.50) and bias-towards-others ($p < .001$; 95% CI, 1.00-1.50). **Discussion:** We improved resident physicians' comfort responding to patient bias-towards-self and bias-towards-others through a toolkit and workshop designed specifically for trainees. The toolkit centers the resident physician perspective, incorporates clinical context, and embeds a debrief. Our novel approach situates the toolkit's teaching in a highly scalable, case-based workshop.

Keywords
Microaggression, Anti-racism, Bias, Case-Based Learning, Communication Skills, Gender Identity, Gender Issues in Medicine, Well-Being/Mental Health, Diversity, Equity, Inclusion

Educational Objectives

By the end of this session, learners will be able to:

1. Recognize the prevalence of experiences of bias among resident physicians.
2. Assess personal emotional bandwidth to determine optimal timing to respond to bias.
3. Utilize debriefing to discuss experiences of bias.
4. Implement toolkit techniques and develop skills through peer-facilitated, simulated cases.
5. Develop increased comfort in responding to bias directed towards themselves or others in clinical care through use of toolkit language.

Citation:
Gay EA, Burnett-Bowie SAM, Bromberg GK. Responding to bias: equipping residents with tools to address microaggressions. *MedEdPORTAL*. 2024;20:11424.
https://doi.org/10.15766/mep_2374-8265.11424

Introduction

Resident physicians frequently experience bias at work.[1] These experiences range from overt racial epithets and sexual harassment to microaggressions, "brief, common indignities that communicate hostile, derogatory or negative slights to persons from minoritized groups."[2] Patients and families have been identified as the most common source of biased statements, with residents from all specialties affected.[2-9] Most female trainees experience gender-based microaggressions, with Latina(x) and Black women especially impacted by gender-based bias.[7,10] Altogether, Asian, Black, Indigenous, and Latinx residents experience more race-based bias than White peers.[11,12] As the medical workforce continues to diversify, more resident physicians may experience patient-expressed bias and/or witness

Copyright © 2024 Gay et al. This is an open-access publication distributed under the terms of the Creative Commons Attribution license.

its effect on colleagues. Many resident physicians report feeling inadequately trained to respond to patient-expressed bias; some trainees report that responding feels futile.[12-14]

Patient-expressed bias contributes to physician burnout.[7,13,15,16] Experiencing burnout harms physicians and negatively impacts the care they provide. Emotional exhaustion and depersonalization, manifestations of burnout associated with experiencing bias, have been linked with increasing reports of medical error and bias towards patients.[15,17] To reduce burnout and adverse clinical outcomes, resident physicians require resources to navigate bias both when responding to bias directed towards them (bias-towards-self) and when (as an ally or upstander) responding to bias directed towards others (bias-towards-others). We aimed to address these needs by developing a 45-minute, hybrid workshop for resident physicians to learn a resident-focused bias-response toolkit and then to practice case-based toolkit implementation. Our toolkit builds on the work of Shankar, Albert, Yee, and Overland, which provides frameworks of pre-, intra-, and post-biased-encounter communication and includes language to use when speaking with the patient and team members.[18] We adapted the intra- and postencounter portions of Shankar and colleagues' framework, streamlining some steps and enhancing focus on trainee emotional response to bias.

Our toolkit and workshop offer a unique trainee-centered strategy to respond to patient-expressed bias. In development, we focused on resident physician well-being by empowering residents to seek support before responding to bias, if needed. Opting for a framework that would validate resident physician identity and experience, we avoided recommending depersonalization.[16] Similarly, we avoided empathy and humor, as we worried about the potential harm done to a target of bias if these strategies were employed by someone else intervening to respond to bias-towards-others. While believing that educating patients to decrease future instances of bias is essential, we did not center on that step in the initial response to bias in our workshop's toolkit. Instead, the perspective is novel in that learner experience and wellness are prioritized over patient education.

Both the toolkit and workshop emphasize team-based responses to bias and incorporate clinical reasoning. Naming clinical contexts where responding to bias must be deferred, either due to patient instability or to altered mental status, acknowledges often unaddressed challenges of frameworks on how to respond to bias in the clinical setting.[14,19] The debrief step, embedded in some toolkits, supports all involved staff in boundary setting and normalizes the idea that these scenarios, like medical emergencies, must be discussed.[18,20,21] Given data that many physicians do not report experienced bias because of concerns about futility or retaliation, frequent debriefs may reduce barriers to responding.[5,13] While other toolkits offer separate language for responding to bias-towards-self versus bias-towards-others, our toolkit can be applied in both scenarios, thus reducing cognitive load and decreasing barriers to implementation. We incorporated cases from our trainees to increase the session's relevance. Workshops like this also increase the awareness of bias in those not affected by a particular type of bias.[22,23]

Although *MedEdPORTAL* has previously published workshops that teach about microaggressions and bias response,[20,24-28] our workshop and toolkit are unique in their resident physician focus. Our workshop is optimized to meet residency educational needs: It is a hybrid, 45-minute session more easily integrated into a busy clinical day for those learners with limited dedicated didactic time. This workshop's small groups are by design self-facilitated to model near-peer problem-solving, enhance psychological safety, and ensure scalability. This approach leverages Bandura's social cognitive theory, where peer learning occurs through observing, retaining, and reproducing demonstrated behavior.[29] Our toolkit is centered on resident physicians' well-being, identifying a personalized optimal time for a trainee to respond to bias, and normalizes opting out of responding if it is not in the trainee's best interest. Finally, our toolkit embeds debriefing to ensure that all bias response is paired with space to process.

Methods

A team of resident and attending physicians developed the workshop and toolkit for a residency curricular thread on diversity, equity, and inclusion. The learners were internal medicine resident physicians, and the goal was to improve comfort in responding to patient-expressed bias towards themselves (bias-towards-self) or patient-expressed bias towards others (bias-towards-others). The workshop was delivered annually during the noontime internal medicine residency educational conference series from 2021 to 2023. The toolkit was also incorporated into the internal medicine residency clinical guide, a handbook used by resident physicians that covered the management of common internal medicine diagnoses, clinical resources, and rotation logistics.

Toolkit Development

Building on approaches to addressing problematic patient behavior developed by Shankar and colleagues,[18] our toolkit

Copyright © 2024 Gay et al. This is an open-access publication distributed under the terms of the Creative Commons Attribution license.

opened with a clinical assessment (Appendix A). Given the toolkit's clinician audience, we expanded this assessment to evaluate both clinical stability and altered mental status. As altered mental status could lead to an unmodifiable source of biased statements, navigating these scenarios might require different actions on the part of the clinical team, including redirecting, frequent debriefing, ignoring bias or changing the subject, and making spaces for impacted team members to excuse themselves.[20,24-26,28] Unlike previously developed approaches to responding to patient-expressed bias, our toolkit employed a required emotional check-in step where the resident physician assessed their emotional reaction to bias and their bandwidth to respond.[14,18,21,30] By normalizing a trainee's assessing their emotional bandwidth before responding to bias, we centered the bias response on the resident physician's needs. These needs could include stepping away from the situation and seeking support before engaging further. If a resident physician chose to engage, we offered a stepwise approach with suggested language to do so. The toolkit was created for response to both bias-towards-self and bias-towards-others to reduce the cognitive load of learning two approaches. Finally, we integrated a debriefing step to standardize both individual and team processing of these events.

Workshop Overview

The workshop occurred during a 45-minute noontime internal medicine residency educational conference and was attended in person or virtually (Table 1). We started the workshop with brief didactics (Appendix B) to avoid prework for busy resident physicians and concluded with case-based toolkit practice in self-facilitated small groups. Facilitators received a facilitator guide to assist in running the workshop (Appendix C). Small-group and self-facilitated discussions were selected to enhance learner psychological safety and for scalability. We chose a case-based approach given its known beneficial impact on improving trainee performance.[31] Virtual participants, who joined via videoconference, were placed in a breakout room or contributed to the case-based practice via the chat function, depending on the number joining virtually.

Pre- and postworkshop survey: Web-based polling software was used, and learners accessed questions via their smartphones. Residents were invited to participate in anonymous pre- and postworkshop surveys (Appendix D).

Introductions: As workshop facilitators, we (Gabrielle Kis Bromberg and Elizabeth A. Gay) opened with personal introductions where we shared our identities and encouraged participant open-mindedness and a growth mindset. We asked attendees to consider their identities, the privilege these identities conferred, and the limitations of their perspectives. We acknowledged the potentially triggering nature of the workshop's content and offered attendees space to disengage as needed, in keeping with the toolkit's aim to remain attentive to one's emotional bandwidth. We established shared definitions of bias, discrimination, microaggressions, and stereotypes to ensure a common vocabulary with participants.

To root the workshop in attendees' experiences and demonstrate the relevance of these skills to trainees, the first polling question was "What names other than 'doctor' have patients called you?" We generated a live word cloud (Figure 1). We chose this prompt because inappropriate terms of endearment, epithets, and misattribution of a resident physician's role were commonly experienced microaggressions.[11,13]

Toolkit review: The didactic portion of the session concluded with a review of the toolkit. There, we shared sample language

Table 1. Workshop Timeline and Sections

Time Allotted	Section
2-5 minutes	Preworkshop survey completion with polling software
5 minutes	Defining bias and sharing data concerning prevalence of bias
5 minutes	Sharing toolkit and best practices for a team-based approach to bias intervention
25 minutes	Practicing with rapid-fire cases alternating the following:<br>● 3 minutes for small-group resident discussion<br>● 5 minutes for large-group, presenter-facilitated reflection
5-8 minutes	Closing didactics including escalation pathway, resource review, and postworkshop survey completion
45 minutes	Total time to complete all sections

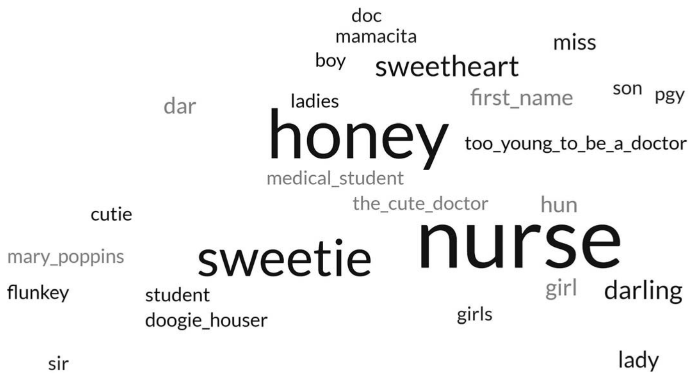

Figure 1. Word cloud generated during the workshop from participant responses to the prompt "What names other than 'doctor' have patients called you?"

Copyright © 2024 Gay et al. This is an open-access publication distributed under the terms of the Creative Commons Attribution license.

developed for each toolkit step with participants (Appendix A). Participants also received a digital copy of the toolkit for reference later in the workshop.

Group discussion, case-based practice, and wrap-up: Participants practiced applying the toolkit to different clinical vignettes. We asked in-person participants to have discussions in groups of three; virtual participants were placed in a breakout room or asked to provide their responses via the chat function. Cases had been created from deidentified resident physician submissions and included some vignettes that consisted of the patient and one resident only and others with a patient and the medical team. Learners practiced applying the toolkit to each case in self-facilitated small groups, with slides featuring the cases displayed at the front of the room (3 minutes). A large-group discussion of each case followed (5 minutes) where facilitators asked each small group to share highlights of its discussion and vignette responses. If attendees were hesitant to share, the facilitators offered previously crafted responses to stimulate discourse (Appendix D). If appropriate, facilitators asked additional questions, including "What makes this scenario challenging?" To highlight the implicit biases participants brought to each scenario, after each case facilitators asked, "What [demographic] assumptions did you make about those depicted in this case?" This process was repeated for each case, generally allowing for discussion of three cases in 25 minutes. To wrap up the session, facilitators shared available residency program, department, and hospital-wide resources for reporting bias, as well as escalation pathways. The toolkit was distributed to attendees at the end of the workshop via email.

Statistical Methods

At our institution, internal medicine residency noontime conference attendance ranged from 20 to 40 persons, with approximately 10% of attendees participating remotely on a videoconferencing platform. We report data collected from 2021 to 2023. Only participants who completed all pre- and postsurvey questions were included in the analyses. Summary statistics of demographic data were calculated as proportions.

We assessed the change in workshop participants' comfort with responding to bias-towards-self and bias-towards-others as measured on a 7-point Likert scale (1 = *very uncomfortable*, 7 = *very comfortable*). The Wilcoxon signed rank test was used to compare pre- versus postworkshop survey responses, and effect size was calculated. Data were stratified by gender and expressed as median and 25th-75th percentiles. All tests were two sided, with $p < .05$ considered statistically significant; there was no correction for multiple comparisons. All statistics were completed in R version 4.2.1 (R Core Team) with RStudio build 2022.07.2+576 (RStudio Team).

Results

Of the 69 attendees who completed the preworkshop survey, 37 (56%) also completed the postworkshop assessment and were included in our analysis. Demographics are shown in Table 2. The workshop increased participants' comfort responding to bias-towards-self ($p < .001$; 95% CI, 1.00-1.50; Figure 2). The effect size was $r = .64$ (95% CI, .39-.83). When stratified by gender, increased comfort in responding to bias-towards-self remained significant for women only ($p < .001$; 95% CI, 1.00-1.50); the effect size was $r = .81$ (95% CI, .69-.88). The workshop also increased comfort responding to bias-towards-others for the overall group ($p < .001$; 95% CI, 1.00-1.50; Figure 2), men ($p = .009$; 95% CI, 1.00-1.50), and women ($p < .001$; 95% CI, 1.00-2.00). The overall effect size was $r = .76$ (95% CI, .61-.86); for men, the effect size was $r = .72$ (95% CI, .39-.92); and for women, it was $r = .79$ (95% CI, .68-.88). A free-text question soliciting workshop feedback received uniformly positive responses. Representative written and verbal attendee feedback after the session included the following:

- "Wonderful, thanks."
- "The most useful part was getting ideas of concrete phrases to use to respond to incidents of bias, something to have ready in a moment of shock."
- "Such an important component of our curriculum on addressing bias and microaggressions."

Table 2. Demographics of Survey Participants ($N = 37$)

Demographic	No. (%)
Gender[a]	
Woman	14 (38)
Man	23 (62)
Training level	
Medical student	3 (8)
Resident	30 (81)
PGY 1	15 (41)
PGY 2	9 (24)
PGY 3	6 (16)
Attending	4 (11)

[a]Gender categories also included nonbinary, other, and prefer not to say; no participant selected these three options, so they are not listed in the table.

Copyright © 2024 Gay et al. This is an open-access publication distributed under the terms of the Creative Commons Attribution license.

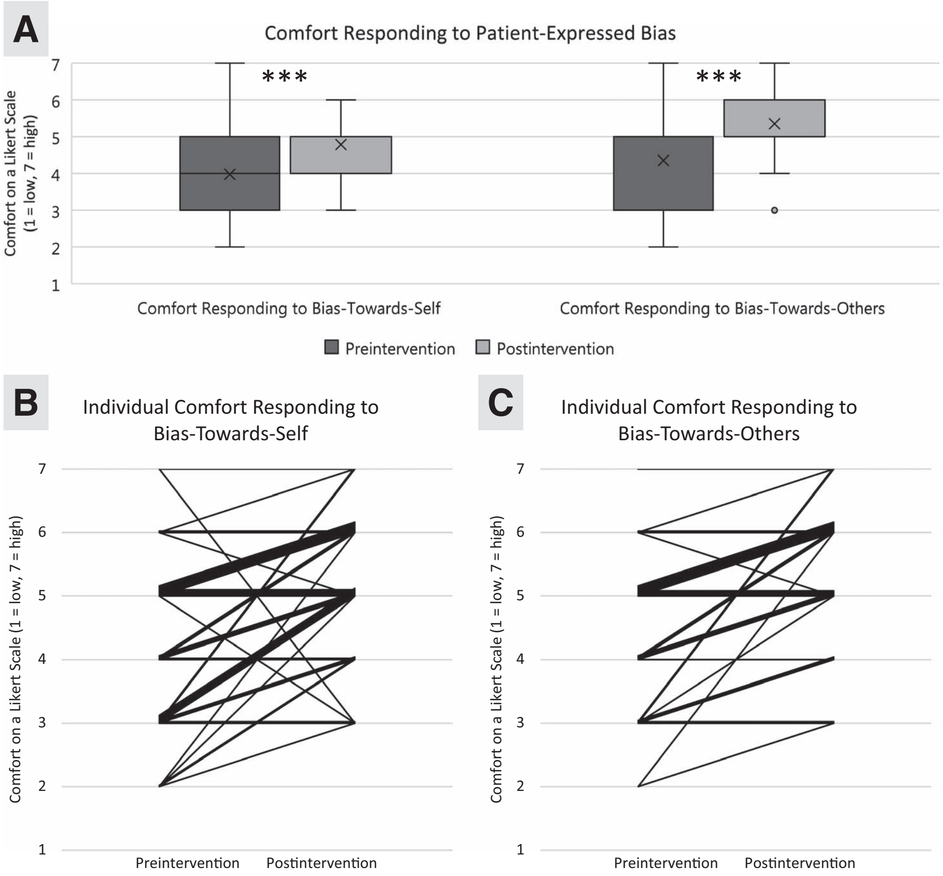

Figure 2. A: Overall summary data for comfort responding to bias-towards-self and bias-towards-others pre- and postintervention presented as first quartile, median, and third quartile, as scored on a 7-point Likert scale (1 = *very uncomfortable*, 7 = *very comfortable*). Significance testing with Wilcoxon signed rank test was significant for both bias-towards-self ($p < .001$; 95% CI, 1.00-1.50) and bias-towards-others ($p < .001$; 95% CI, 1.00-1.50). The bars demonstrate the ranges of the first and fourth quartiles. The shaded areas are the second and third quartiles, with the horizontal bar being the median (not visible on all boxplots due to tighter clustering of scores, hence the Xs as mean markers). The asterisks (***) indicate level of significance between pre- and postintervention scores (<.001). The open circle is an outlier point. B: Participant comfort responding to bias-towards-self represented with individual Likert scores pre- and postintervention. C: Participant comfort responding to bias-towards-others represented with individual Likert scores pre- and postintervention.

Discussion

We developed a 45-minute workshop that equipped resident physicians with trainee-tailored tools to respond to patient-expressed bias and microaggressions. The workshop significantly improved resident physicians' comfort in addressing bias directed towards themselves and towards others. It increased women participants' comfort in responding to both types of bias; however, it increased men participants' comfort in responding to bias-towards-others, only.

Though this workshop could have been debuted with other learner populations (attending physicians, nurse practitioners, physician assistants, nurses, or other members of the health care workforce), we focused on resident physicians first because of their vulnerable status within academic medicine. Residents are constrained by the evaluative nature of training programs, specifically, the fear that acknowledging bias could result in negative evaluations of professional competency.[13] Furthermore, professional identity development and sense of belonging may be especially harmed by experiences of bias.[12,13,15,16] We believe that encouraging assessment of one's emotional state and/or bandwidth is particularly important for trainees when navigating difficult conversations. We have successfully given this same workshop (unchanged) to other learner populations at our institution.

Finding protected, nonclinical time for resident physician teaching is challenging. To address this, we embedded the workshop in a preexisting hybrid conference to maximize resident access. This workshop could easily be adapted to an in-person-only or remote-only setting without affecting its impact.[9] A near-peer facilitated workshop model ensures trainees are the

Copyright © 2024 Gay et al. This is an open-access publication distributed under the terms of the Creative Commons Attribution license.

primary drivers of their own growth, optimizes psychological safety, encourages development of shared language and debriefing community, and guarantees scalability in workshop delivery to variably sized residencies without reliance on facilitators.

Limitations of this workshop include the following. To increase psychological safety, we did not collect race and ethnicity data. More residents attended the workshop than completed the pre- and postworkshop assessments, likely due to competing patient care duties. We did not assess whether the impact of the workshop differed based on in-person versus virtual participation. Finally, given that the workshop was conducted at one program in a single institution, generalizability is unclear.

Future goals include assessing the durability of participants' increased comfort in responding to bias to better understand the optimal frequency of future workshops and assessing potential impact in other specialties and learner populations. Though we have shown this toolkit and workshop to be effective in increasing resident physician comfort in responding to patient-expressed bias, better understanding the toolkit's value for responding to bias expressed by other members of the health care team is key. As this is a hybrid workshop, future study should examine whether there is a difference in impact for in-person compared to virtual participants. While understanding learner comfort is essential, it remains a proxy for behavioral change. In the long run, increasing individuals' intervention in bias-towards-others (upstander or allyship behavior) will be an important step in achieving culture change in medicine to promote inclusion. Based on the ease of implementing the workshop's near-peer, self-facilitated structure, we recommend its use across graduate medical education and believe it can also be successfully leveraged by clinical faculty. Overall, our toolkit offers a novel, clinically relevant, and resident physician-centered approach and aims to embed debriefing in the culture. The toolkit was effectively deployed as the main educational tool in a workshop that yielded a significant increase in trainee comfort responding to patient-expressed bias.

Appendices

A. Bias Response Toolkit.docx

B. Bias Response Workshop.pptx

C. Facilitator Guide.docx

D. Pre- and Postworkshop Survey Questions.docx

All appendices are peer reviewed as integral parts of the Original Publication.

Elizabeth A. Gay, MD: Third-Year Resident, Department of Medicine, Massachusetts General Hospital; ORCID: https://orcid.org/0000-0002-7151-5082

Sherri-Ann M. Burnett-Bowie, MD, MPH: Associate Professor of Medicine, Endocrine Division, Department of Medicine, Massachusetts General Hospital and Harvard Medical School; ORCID: https://orcid.org/0000-0002-0064-096X

Gabrielle Kis Bromberg, MD: Instructor of Medicine and Hospitalist, Department of Medicine, Massachusetts General Hospital and Harvard Medical School; ORCID: https://orcid.org/0000-0002-2140-7702

Disclosures
None to report.

Funding/Support
None to report.

Ethical Approval
Reported as not applicable.

References

1. Bahji A, Altomare J. Prevalence of intimidation, harassment, and discrimination in resident physicians: a systematic review and meta-analysis. *Can Med Educ J*. 2020;11(1):e97-e123. https://doi.org/10.36834/cmej.57019
2. Turner J, Higgins R, Childs E. Microaggression and implicit bias. *Am Surg*. 2021;87(11):1727-1731. https://doi.org/10.1177/00031348211023418
3. Mustapha T, Ho Y, Andrews JS, Cullen MJ. See no evil, hear no evil, stop no evil: institutional-level tracking to combat mistreatment of residents and fellows. *J Grad Med Educ*. 2019;11(5):601-605. https://doi.org/10.4300/JGME-D-19-00218.1
4. Schlick CJR, Ellis RJ, Etkin CD, et al. Experiences of gender discrimination and sexual harassment among residents in general surgery programs across the US. *JAMA Surg*. 2021;156(10):942-952. https://doi.org/10.1001/jamasurg.2021.3195
5. Wang LJ, Tanious A, Go C, et al. Gender-based discrimination is prevalent in the integrated vascular trainee experience and serves as a predictor of burnout. *J Vasc Surg*. 2020;71(1):220-227. https://doi.org/10.1016/j.jvs.2019.02.064

Copyright © 2024 Gay et al. This is an open-access publication distributed under the terms of the Creative Commons Attribution license.

6. Gadjradj PS, Ghobrial JB, Booi SA, de Rooij JD, Harhangi BS. Mistreatment, discrimination and burn-out in neurosurgery. *Clin Neurol Neurosurg*. 2021:202:106517. https://doi.org/10.1016/j.clineuro.2021.106517

7. Kemper KJ, Schwartz A; Pediatric Resident Burnout-Resilience Study Consortium. Bullying, discrimination, sexual harassment, and physical violence: common and associated with burnout in pediatric residents. *Acad Pediatr*. 2020;20(7):991-997. https://doi.org/10.1016/j.acap.2020.02.023

8. Lall MD, Bilimoria KY, Lu DW, et al. Prevalence of discrimination, abuse, and harassment in emergency medicine residency training in the US. *JAMA Netw Open*. 2021;4(8):e2121706. https://doi.org/10.1001/jamanetworkopen.2021.21706

9. Bromberg GK, Gay EA, Hills-Dunlap K, Burnett-Bowie SAM. Using a virtual platform to teach residents how to respond to bias. *J Gen Intern Med*. 2022;37(11):2871-2872. https://doi.org/10.1007/s11606-021-07156-y

10. McKinley SK, Wang LJ, Gartland RM, et al. “Yes, I’m the doctor”: one department’s approach to assessing and addressing gender-based discrimination in the modern medical training era. *Acad Med*. 2019;94(11):1691-1698. https://doi.org/10.1097/ACM.0000000000002845

11. Osseo-Asare A, Balasuriya L, Huot SJ, et al. Minority resident physicians’ views on the role of race/ethnicity in their training experiences in the workplace. *JAMA Netw Open*. 2018;1(5): e182723. https://doi.org/10.1001/jamanetworkopen.2018.2723

12. de Bourmont SS, Burra A, Nouri SS, et al. Resident physician experiences with and responses to biased patients. *JAMA Netw Open*. 2020;3(11):e2021769. https://doi.org/10.1001/jamanetworkopen.2020.21769

13. Wheeler M, de Bourmont S, Paul-Emile K, et al. Physician and trainee experiences with patient bias. *JAMA Intern Med*. 2019;179(12):1678-1685. https://doi.org/10.1001/jamainternmed.2019.4122

14. Whitgob EE, Blankenburg RL, Bogetz AL. The discriminatory patient and family: strategies to address discrimination towards trainees. *Acad Med*. 2016;91(11)(suppl):S64-S69. https://doi.org/10.1097/ACM.0000000000001357

15. Dyrbye L, Herrin J, West CP, et al. Association of racial bias with burnout among resident physicians. *JAMA Netw Open*. 2019; 2(7):e197457. https://doi.org/10.1001/jamanetworkopen.2019.7457

16. Dyrbye LN, West CP, Sinsky CA, et al. Physicians’ experiences with mistreatment and discrimination by patients, families, and visitors and association with burnout. *JAMA Netw Open*. 2022;5(5):e2213080. https://doi.org/10.1001/jamanetworkopen.2022.13080

17. West CP, Tan AD, Habermann TM, Sloan JA, Shanafelt TD. Association of resident fatigue and distress with perceived medical errors. *JAMA*. 2009;302(12):1294-1300. https://doi.org/10.1001/jama.2009.1389

18. Shankar M, Albert T, Yee N, Overland M. Approaches for residents to address problematic patient behavior: before, during, and after the clinical encounter. *J Grad Med Educ*. 2019;11(4): 371-374. https://doi.org/10.4300/JGME-D-19-00075.1

19. Paul-Emile K, Smith AK, Lo B, Fernández A. Dealing with racist patients. *N Engl J Med*. 2016;374(8):708-711. https://doi.org/10.1056/NEJMp1514939

20. Neves da Silva HV, Heery LM, Cohen WR, et al. What happened and why: responding to racism, discrimination, and microaggressions in the clinical learning environment. *MedEdPORTAL*. 2022;18:11280. https://doi.org/10.15766/mep_2374-8265.11280

21. Fisher HN, Chatterjee P, Shapiro J, Katz JT, Yialamas MA. “Let’s talk about what just happened”: a single-site survey study of a microaggression response workshop for internal medicine residents. *J Gen Intern Med*. 2021;36(11):3592-3594. https://doi.org/10.1007/s11606-020-06576-6

22. Wilker CG, Stockham AL, Houge BJ, Stevens SK, Munson KA, Mueller PS. Computer-based patient bias and misconduct training impact on reports to incident learning system. *Mayo Clin Proc Innov Qual Outcomes*. 2021;5(6):1075-1080. https://doi.org/10.1016/j.mayocpiqo.2021.08.013

23. Periyakoil VS, Chaudron L, Hill EV, Pellegrini V, Neri E, Kraemer HC. Common types of gender-based microaggressions in medicine. *Acad Med*. 2020;95(3):450-457. https://doi.org/10.1097/ACM.0000000000003057

24. DallaPiazza M, Padilla-Register M, Dwarakanath M, Obamedo E, Hill J, Soto-Greene ML. Exploring racism and health: an intensive interactive session for medical students. *MedEdPORTAL*. 2018;14:10783. https://doi.org/10.15766/mep_2374-8265.10783

25. Acholonu RG, Cook TE, Roswell RO, Greene RE. Interrupting microaggressions in health care settings: a guide for teaching medical students. *MedEdPORTAL*. 2020;16:10969. https://doi.org/10.15766/mep_2374-8265.10969

26. Sandoval RS, Afolabi T, Said J, Dunleavy S, Chatterjee A, Ölveczky D. Building a tool kit for medical and dental students: addressing microaggressions and discrimination on the wards. *MedEdPORTAL*. 2020;16:10893. https://doi.org/10.15766/mep_2374-8265.10893

27. Walker VP, Hodges L, Perkins M, Sim M, Harris C. Taking the VITALS to interrupt microaggressions. *MedEdPORTAL*. 2022;18:11202. https://doi.org/10.15766/mep_2374-8265.11202

28. Ackerman-Barger K, Jacobs NN, Orozco R, London M. Addressing microaggressions in academic health: a workshop for inclusive excellence. *MedEdPORTAL*. 2021;17:11103. https://doi.org/10.15766/mep_2374-8265.11103

29. Zackoff MW, Real FJ, Abramson EL, Li STT, Klein MD, Gusic ME. Enhancing educational scholarship through conceptual frameworks: a challenge and roadmap for medical educators.

Copyright © 2024 Gay et al. This is an open-access publication distributed under the terms of the Creative Commons Attribution license.

Acad Pediatr. 2019;19(2):135-141. https://doi.org/10.1016/j.acap.2018.08.003

30. Stevens SK, Houge BJ, Poterucha JM, et al. Implementing SAFER responses to misconduct and responding to biased patient requests ASAP. *J Patient Exp*. 2022;9:23743735221102672. https://doi.org/10.1177/23743735221102672
31. Sartania N, Sneddon S, Boyle JG, McQuarrie E, de Koning HP. Increasing collaborative discussion in case-based learning improves student engagement and knowledge acquisition. *Med Sci Educ*. 2022;32(5):1055-1064. https://doi.org/10.1007/s40670-022-01614-w

Received: August 3, 2023
Accepted: March 22, 2024
Published: August 6, 2024

Copyright © 2024 Gay et al. This is an open-access publication distributed under the terms of the Creative Commons Attribution license.
